# Structural basis of meiotic chromosome synaptic elongation through hierarchical fibrous assembly of SYCE2-TEX12

**DOI:** 10.1038/s41594-021-00636-z

**Published:** 2021-08-09

**Authors:** James M. Dunce, Lucy J. Salmon, Owen R. Davies

**Affiliations:** 1 Wellcome Centre for Cell Biology, Institute of Cell Biology, University of Edinburgh, Michael Swann Building, Max Born Crescent, Edinburgh EH9 3BF, UK; 2 Biosciences Institute, Faculty of Medical Sciences, Newcastle University, Framlington Place, Newcastle upon Tyne NE2 4HH, UK

**Keywords:** Meiosis, chromosome structure, double-strand break, chiasmata, synaptonemal complex, SYCE2, TEX12, self-assembly, coiled-coil, small-angle X-ray scattering, biophysics, X-ray crystallography, intermediate filaments

## Abstract

The synaptonemal complex (SC) is a supramolecular protein assembly that mediates synapsis between homologous chromosomes during meiosis. SC elongation along the chromosome length (up to 24 μm) depends on its midline α-fibrous component SYCE2-TEX12. Here, we report X-ray crystal structures of human SYCE2-TEX12 as an individual building-block and upon assembly within a fibrous lattice. We combine these structures with mutagenesis, biophysics and electron microscopy to reveal the hierarchical mechanism of SYCE2-TEX12 fibre assembly. SYCE2-TEX12’s building-blocks are 2:2 coiled-coils which dimerise into 4:4 hetero-oligomers and interact end-to-end and laterally to form 10-nm fibres, which intertwine within 40-nm bundled micrometre-long fibres that define the SC’s midline structure. This assembly mechanism bears striking resemblance with intermediate filament proteins vimentin, lamin and keratin. Thus, SYCE2-TEX12 exhibits behaviour typical of cytoskeletal proteins to provide an α-fibrous SC backbone that structurally underpins synaptic elongation along meiotic chromosomes.

## Introduction

Meiosis, the specialised form of cell division in gametogenesis, involves an extraordinary chromosome choreography that generates haploid oocytes and spermatozoa^1^. The hallmark of meiosis is synapsis, in which aligned homologous chromosomes become tightly bound along their axial length whilst undergoing recombination and crossover formation^1^. This is achieved by the synaptonemal complex (SC), a supramolecular protein assembly that acts as a molecular ‘zipper’ of meiotic chromosomes^1,2^. SC integrity is essential for meiosis^3^, and mutations that disrupt SC structure have been identified in clinical cases of infertility^4,5^. Thus, the mechanism of SC assembly is fundamentally important for understanding human fertility and the molecular causes of infertility, miscarriage and aneuploidy.

In advance of synapsis, homologous chromosome pairs are established and aligned by recombination intermediates generated from 200-400 induced double-strand breaks per cell^1^. Concomitant with recombination, chromosomes adopt a ‘lamp-brush’ structure, in which chromatin is looped around linear axes^6,7^. Thus, the SC assembles between aligned linear arrays of homologous chromatin and converts discrete recombination-mediated links into a single continuous synapsis along the axial length. The SC’s assembled three-dimensional structure facilitates the resolution of recombination intermediates, forming typically one crossover per arm^3,8^. In addition to providing genetic diversity, crossovers provide the sole physical connections between homologues following SC disassembly, thereby enabling faithful homologous chromosome segregation at the end of meiosis I^1,2^.

**Fig. 1:**
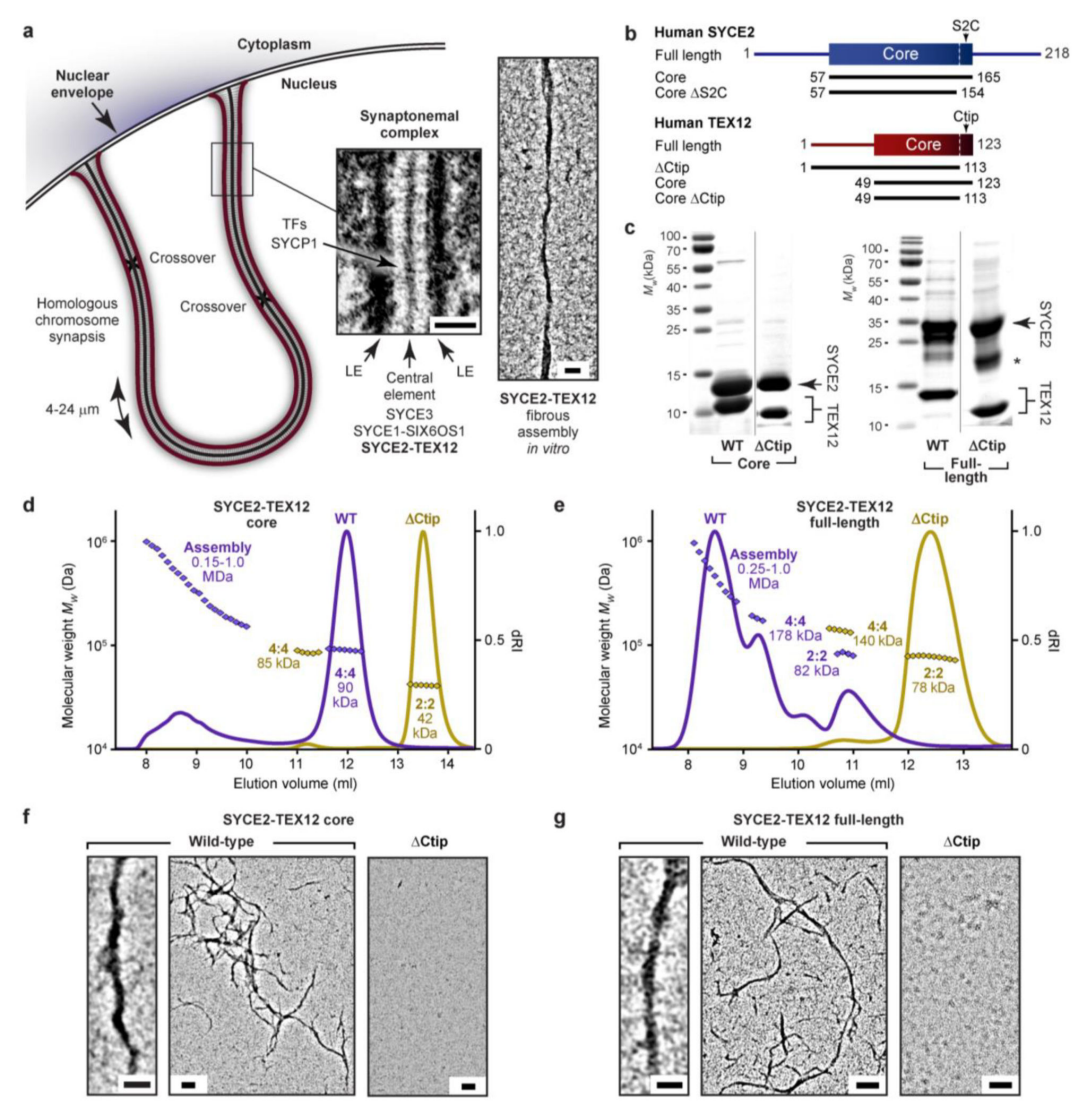
SYCE2-TEX12 self-assembly is driven by the C-terminal tip of TEX12. (**a**) Schematic of the synaptonemal complex (SC) mediating full synapsis between homologous chromosomes that are physically tethered at both ends to the nuclear envelope. The SC has a tripartite structure of an electron-dense central element lying midway between chromosome-bound lateral elements (electron micrograph reproduced from Kouznetsova, et al.^3^). SC central element formation depends on an SYCE2-TEX12 complex that undergoes fibrous self-assembly *in vitro* (electron micrograph reproduced from Davies, et al. ^31^). Scale bars, 100 nm. (b) Human SYCE2 and TEX12 sequences, depicting their α-helical structural cores and the principal constructs used in this study. (c) SDS-PAGE of purified SYCE2-TEX12 full-length and core complexes containing wild-type (WT) and ΔCtip TEX12 sequences. The dominant degradation product of SYCE2 is indicated by an asterisk. Representative of at least three independent experiments. (d,e) SEC-MALS analysis of SYCE2-TEX12 (d) core and (e) full-length complexes in which differential refractive index (dRI) is shown with fitted molecular weights (*Mw*) plotted as diamonds across elution peaks. (d) SYCE2-TEX12 core forms a 90 kDa 4:4 complex (75% of total mass; theoretical *Mw* - 89 kDa) and large molecular assemblies of up to 1.0 MDa (25%). Truncation of TEX12’s C-terminal tip (ΔCtip) restricts SYCE2-TEX12 assembly to a 42 kDa 2:2 complex (98%; theoretical - 42 kDa) and 85 kDa 4:4 complex (2%; theoretical – 84 kDa). Samples were analysed at 200 μM (4 mg/ml). (e) SYCE2-TEX12 full-length forms an 82 kDa 2:2 complex (12%; theoretical – 78 kDa), 178 kDa 4:4 complex (18%; theoretical – 156 kDa) and large molecular assemblies of up to 1.0 MDa (70%). The TEX12 ΔCtip truncation restricts SYCE2-TEX12 assembly to a 78 kDa 2:2 complex (98%; theoretical – 76 kDa) and 140 kDa 4:4 complex (2%; theoretical – 151 kDa). Samples were analysed at 100 μM (4 mg/ml). (f) Electron micrographs of SYCE2-TEX12 core wild-type and ΔCtip. Scale bars, 50 nm (left) and 100 nm (middle and right). (g) Electron micrographs of SYCE2-TEX12 full-length wild-type and ΔCtip. Scale bars, 25 nm (left) and 100 nm (middle and right). (f,g) Representative of at least three independent experiments.

The SC was discovered in 1956 through its iconic tripartite appearance on electron micrographs, initially in crayfish spermatocytes^9^, and subsequently throughout mammals, insects, plants and yeast^10,11^. The SC’s tripartite electron-dense fibres correspond to the two chromosome axes (lateral elements), separated by 100-nm, with a midline central element (Fig. 1a)^10,11^. Central and lateral elements have widths of approximately 20-40 nm and 50 nm, respectively, are continuous along the chromosome length (4-24 μm in humans), and are held together by interdigitated transverse filaments that act as the teeth of the SC ‘zipper’^10–14^. Electron microscopy and super-resolution fluorescence microscopy have shown that the mammalian SC has a depth of up to 100 nm^13,15,16^. Assuming a square cross-section of 100-nm sides, with 20-80% solvent content, we can estimate a molecular weight of 1.6-6.4 GDa per μm of SC length. Thus, entire SCs may be between 6.4-154 GDa, placing them amongst the largest protein structures within a cell.

Over the last thirty years, protein components of the mammalian SC have been identified and their roles defined by genetics and cellular imaging. Transverse filaments are formed by SYCP1 molecules, which are bioriented with their N- and C-termini within central and lateral elements, respectively, such that opposing molecules span the SC’s 100-nm width^13,16,17^. Lateral elements contain SYCP2-3^18–20^, whilst the central element is formed of SYCE1-3, TEX12 and SIX6OS1^21–24^. SYCP1 and central element proteins are essential for SC formation, crossover formation and meiosis^22,24–28^. However, there are important differences between their knockout phenotypes. The SYCP1 knockout exhibits complete synaptic failure with no central element recruitment^22,24,25,28^. In SYCE1, SYCE3 and SIX6OS1 knockouts, there is patchy SYCP1 recruitment, with some chromosomal associations but no tripartite SC^22,24,26^. In contrast, SYCE2 and TEX12 knockouts demonstrate short tripartite structures, containing SYCP1, SYCE1, SYCE3 and SIX6OS1, which fail to extend along the chromosome axis^27,28^. Thus, it has been proposed that SYCE1, SYCE3 and SIX6OS1 are initiation factors that provide short-range stabilisation of nascent SYCP1 synapsis, whereas SYCE2 and TEX12 are elongation factors that underpin long-range SC growth by providing longitudinal structural support^22,27–29^. This dichotomy is underpinned by biochemical findings that SYCE1 directly interacts with SYCE3 and SIX6OS^14,30^, whilst SYCE2 and TEX12 exist in a seemingly constitutive complex^31^.

In the last decade, the molecular underpinnings of the SC have started to come into focus through analysis of SC proteins by structural biology^4,17,30–37^. An emerging theme is that SC proteins, which are almost universally coiled-coils, exist as defined building-blocks that self-assemble into higher-order structures to provide the SC’s distinct architectural elements. SYCP1 is a tetramer that assembles into a lattice-like array to fulfil the SC’s fundamental role of tethering chromosome axes^17^. SYCP3 is a tetramer that assembles into paracrystalline fibres, which have been observed for recombinant proteins *in vitro* ^32,37^, upon heterologous cellular expression^38^, and within the native SC^39^. SYCP3 fibres separate DNA-binding sites by 23-nm repeating units, facilitating the compaction of chromatin loops along the meiotic chromosome axis^32^. Similar structures and assemblies are achieved by an SYCP2-SYCP3 complex^36^. Central element proteins SYCE2 and TEX12 form a core 4:4 complex that assembles into fibres with polymorphic appearance, of 40-nm width, and up to 5-μm in length (Fig. 1a)^31^. The appearance and dimensions of SYCE2-TEX12 fibres match those of the native SC central element, suggesting that fibrous assembly along the SC axis underlies the structural role of SYCE2-TEX12 in synaptic elongation.

Here, we combine X-ray crystal structures of SYCE2-TEX12 at two critical stages of assembly with solution and electron microscopy studies to reveal a hierarchical mechanism for SYCE2-TEX12 α-fibre assembly, which intriguingly resembles intermediate filament assembly. The building blocks of SYCE2-TEX12 assembly are 2:2 complexes, which dimerise into 4:4 hetero-octamers that undergo end-to-end and staggered lateral interactions to form 10-nm fibres. These thread-like structures become intertwined within 40-nm fibres that extend to several micrometres in length and resemble the native SC central element. Thus, we define the molecular mechanism whereby SYCE2-TEX12 forms an α-fibrous axis that provides long-range structural support for synapsis as the ‘backbone’ of the SC.

## Results

### SYCE2-TEX12 assembly is mediated by TEX12’s C-terminal tip

**Extended Data Fig. 1:**
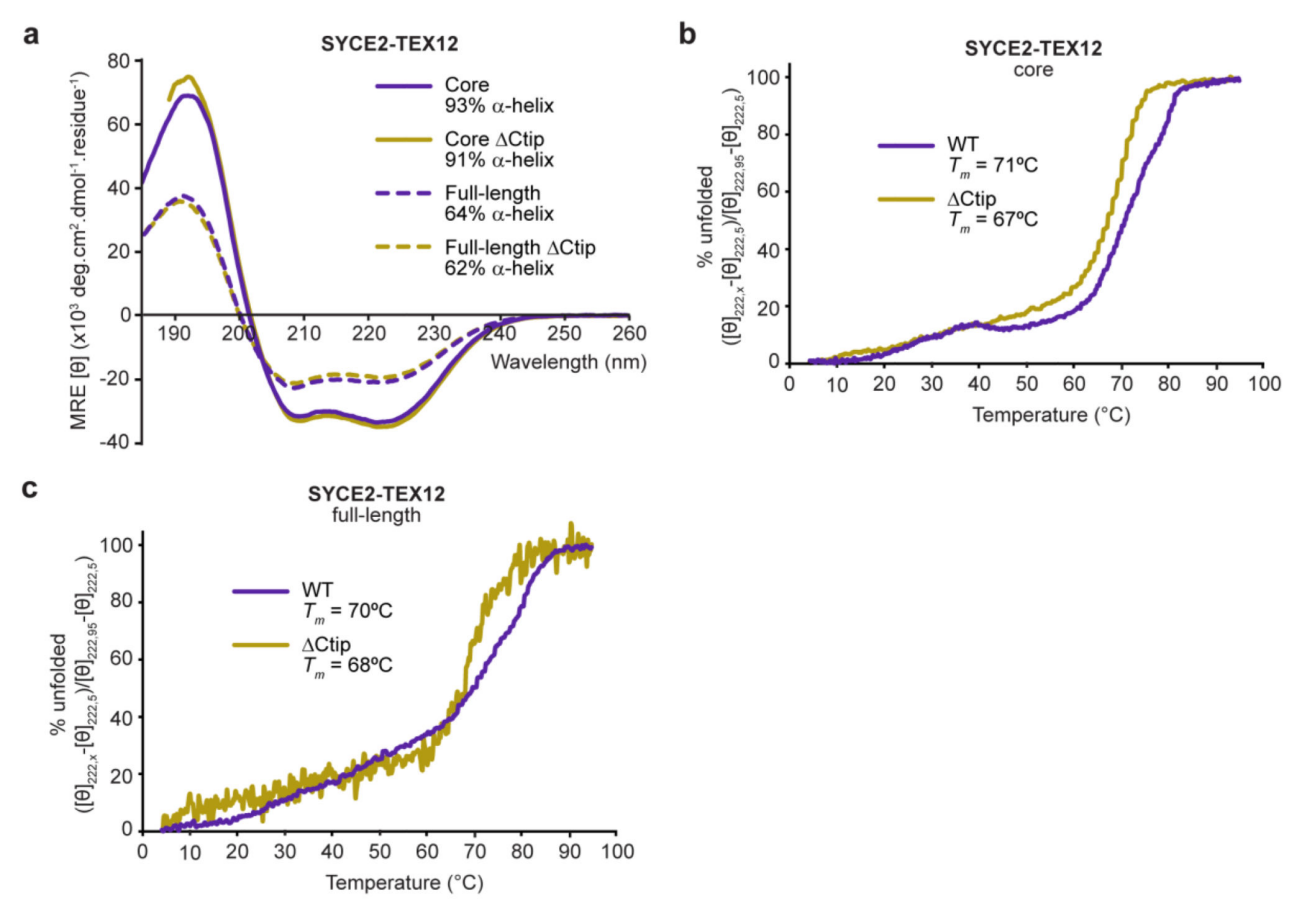
Circular dichroism analysis of SYCE2-TEX12 complexes. (**a-c**) Circular dichroism (CD) analysis of SYCE2-TEX12 core and full-length complexes containing wild-type (WT; purple) and ΔCtip (yellow) TEX12 sequences. (**a**) Far UV circular dichroism (CD) spectra recorded between 260 nm and 185 nm in mean residue ellipticity, MRE ([θ]) (x10^3^ deg.cm^2^.dmol^−1^.residue^−1^). Data were deconvoluted using the CDSSTR algorithm, with helical content indicated. (**b-c**) CD thermal denaturation of SYCE2-TEX12 (**b**) core and (**c**) full-length complexes, recording the CD helical signature at 222 nm between 5°C and 95°C, as % unfolded. Melting temperatures were estimated, as indicated.

A common feature of self-assembly by SC proteins SYCP1-3 is the presence of short motifs at their α-helical ends that direct higher-order assembly^17,32,36^. In each case, disruption of these motifs blocks assembly and restricts proteins to obligate ‘building block’ structures. Could a similar phenomenon apply to SYCE2-TEX12 fibres? To test this, we analysed the oligomeric state and assembly of short deletions of the SYCE2-TEX12 core (amino-acids 57-165 and 49-123; Fig. 1b) by size-exclusion chromatography multi-angle light scattering (SEC-MALS). Having confirmed that wild-type SYCE2-TEX12 core is a 4:4 complex that forms higher-order structures (Fig. 1c,d), we identified that deletion of TEX12’s C-terminal tip (ΔCtip, amino-acids 49-113; Fig. 1b) restricted SYCE2-TEX12 to a 2:2 complex and blocked higher-order assembly (Fig. 1c,d). Circular dichroism (CD) determined that wild-type and ΔCtip complexes have comparable α-helical contents (93% and 91%) and melting temperatures (71°C and 67°C) (Extended Data Fig. 1a,b). Thus, the ΔCtip 2:2 complex may constitute a substructure of the wild-type core 4:4 complex that represents its minimum building block for assembly.

Full-length SYCE2-TEX12 has an increased propensity for assembling in solution, with SEC-MALS revealing its predominant formation of higher-order species, in addition to clearly discernible 4:4 and 2:2 complexes (Fig. 1c,e). The presence of a wild-type 2:2 complex supports its putative role as an obligate building block. In common with the core complex, deletion of TEX12’s C-terminal tip restricted full-length SYCE2-TEX12 to a 2:2 complex and blocked higher-order assembly (Fig. 1c,e). The α-helical structure and stability of the wild-type protein was retained in the ΔCtip complex (Extended Data Fig. 1a,c). Thus, in a manner reminiscent of SYCP1-3^17,32,36^, deletion of a short motif at TEX12’s C-terminal tip blocks higher-order assembly and stabilises SYCE2-TEX12 in its building-block 2:2 complex.

**Extended Data Fig. 2:**
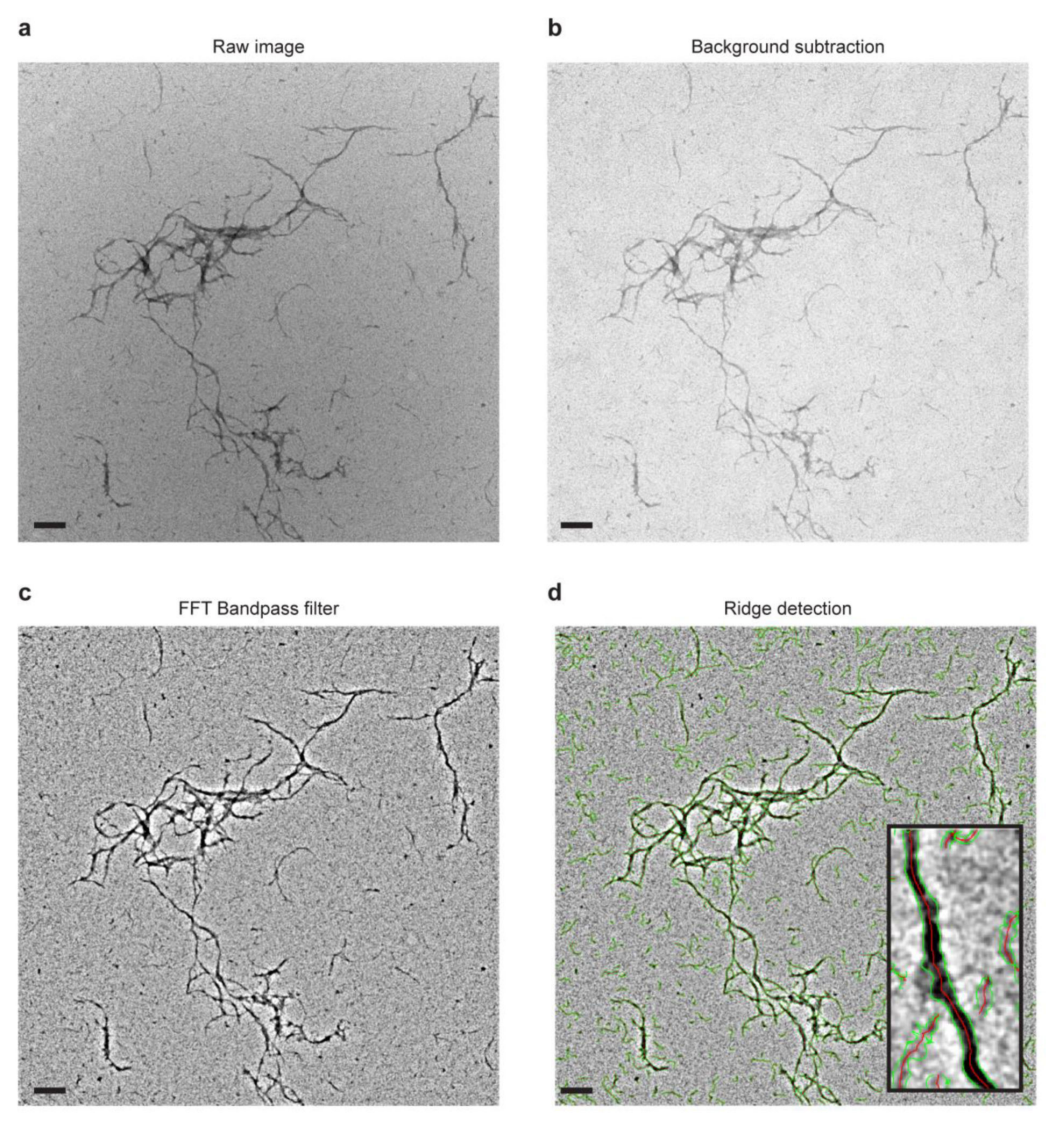
Image analysis of SYCE2-TEX12 electron micrographs. (**a-d**) The procedure used for automated image analysis and quantification of SYCE2-TEX12 fibres is shown for an example micrograph. The Fiji distribution of ImageJ was used to process (**a**) raw micrographs through (**b**) background subtraction and (**c**) FFT Bandpass filter. (**d**) Fibres were detected by the Ridge detection algorithm in which the centre and edges of interpreted fibres were highlightedin red and green, respectively, from which mean fibre widths and fibre lengths were determined. Representative of at least three independent experiments.

**Extended Data Fig. 3:**
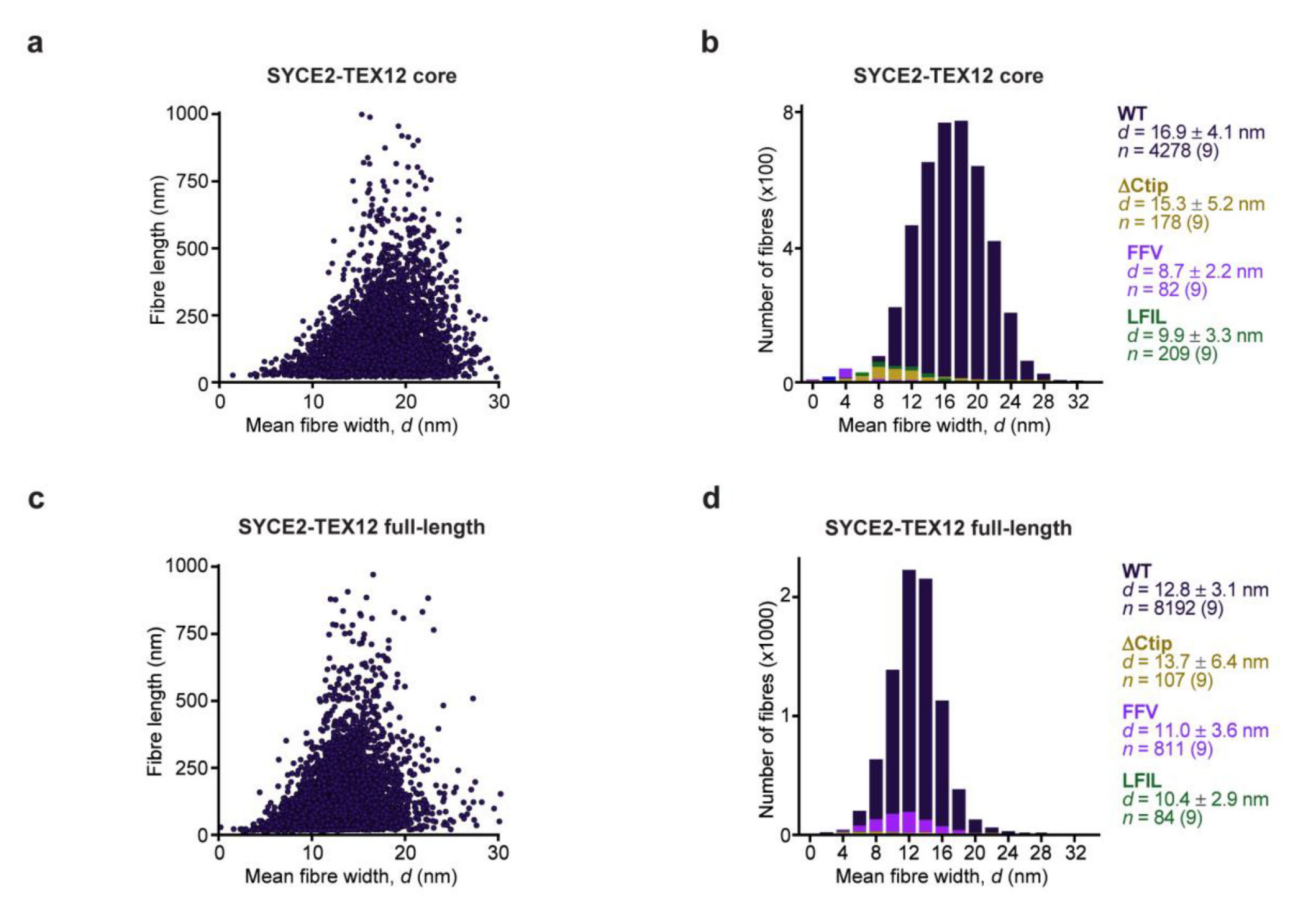
Electron microscopy analysis of SYCE2-TEX12. (**a-d**) Electron microscopy of SYCE2-TEX12 (**a,b**) core and (**c,d**) wild-type, relating to Fig. 1f,g. (**a**) Scatter plot of mean fibre width (*d,* nm) against fibre length (nm) for wild-type SYCE2-TEX12 core. (**b**) Histograms of the number of fibres with mean widths within 2-nm bins for SYCE2-TEX12 core wild-type (dark blue), ΔCtip (yellow), FFV (F102A, F109A, V116A; purple) and LFIL (L110E, F114E, I117E, L121E; green). The mean, standard deviation and number of fibres in each population is shown, each determined from nine micrographs. (c) Scatter plot of mean fibre width (d, nm) against fibre length (nm) for wild-type SYCE2-TEX12. (d) Histograms of the number of fibres with mean widths within 2nm bins for SYCE2-TEX12 full-length wild-type (dark blue), ΔCtip (yellow), FFV (light blue) and LFIL (green). The mean, standard deviation and number of fibres in each population is shown, each determined from nine micrographs.

We next visualised SYCE2-TEX12 assembly by electron microscopy (Fig. 1f,g). This confirmed that core and full-length complexes form morphologically similar fibres, which are typically positively stained, vary in thickness up to approximately 40 nm and extend up to several micrometres in length (Fig. 1f,g). Their distributions were determined by automated detection and measurement (Extended Data Figs. 2 and 3), revealing that despite their similar lengths, mean fibre widths were slightly smaller for the full-length complex (12.8 ± 3.1 nm; Extended Data Fig. 3c,d) than its structural core (16.9 ± 4.1 nm; Extended Data Fig. 3a,b). This suggests that flanking sequences provide additional stability to core assemblies. In agreement with SEC-MALS, TEX12 ΔCtip blocked fibre formation of core and full-length complexes (Fig. 1f,g and Extended Data Fig. 3b,d). We conclude that TEX12’s C-terminal tip is essential for assembly of SYCE2-TEX12 fibres, and its deletion stabilises the building-block 2:2 structure, providing a critical means for elucidating the fibre assembly mechanism.

### Crystal structure of SYCE2-TEX12 in a 2:2 complex

**Extended Data Fig. 4:**
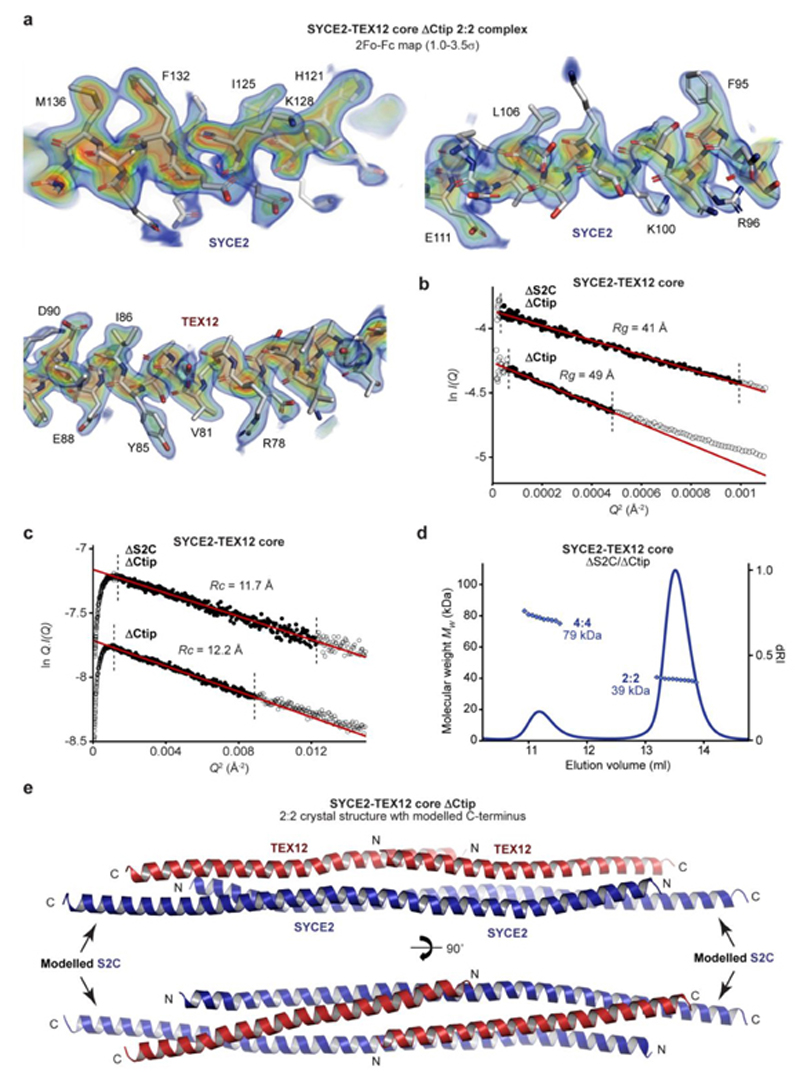
Crystal structure of the SYCE2-TEX12 core 2:2 complex. (**a**) 2Fo-Fc electron density map of the SYCE2-TEX12 core ΔCtip 2:2 complex, presented as a rainbow between 1.0σ (blue) and 3.5σ (red), superimposed on the refined crystallographic model. (**b-c**) SEC-SAXS analysis of SYCE2-TEX12 core ΔS2C/ΔCtip. (**b**) SAXS Guinier analysis to determine the radius of gyration (*Rg*); linear fits are shown in red, with the fitted data range highlighted in black and demarcated by dashed lines. The *Q.Rg* values were < 1.3 and *Rg* was calculated as 41 Å and 49 Å, respectively. (**c**) SAXS Guinier analysis to determine the radius of gyration of the cross-section (Rc); linear fits are shown in red, with the fitted data range highlighted in black and demarcated by dashed lines. The *Q.Rc* values were < 1.3 and *Rc* was calculated as 11.7 Å and 12.2 Å, respectively. (**d**) SEC-MALS analysis of SYCE2-TEX12 core ΔS2C/ΔCtip (dRI profile with molecular weights plotted as diamonds) showing the formation of a 39 kDa 2:2 complex (86%; theoretical – 40 kDa) and a 79 kDa 4:4 complex (14%; theoretical – 79 kDa). (e) Theoretical model of the full SYCE2-TEX12 core ΔCtip 2:2 complex in which the missing C-terminal coiled-coil and S2C helix were docked onto the crystal structure.

**Fig. 2:**
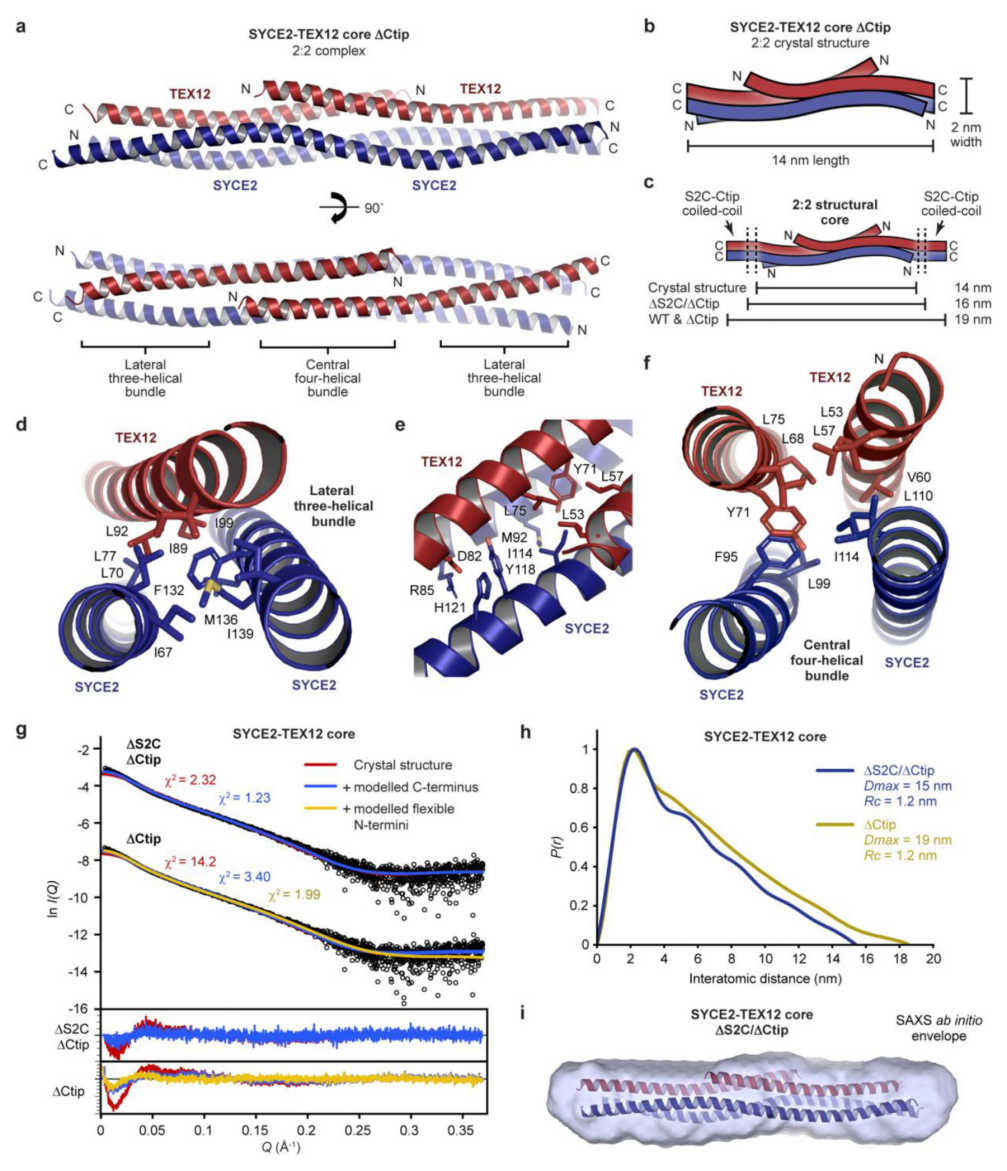
Crystal structure of the SYCE2-TEX12 core 2:2 complex. (**a**) Crystal structure of SYCE2-TEX12 core ΔCtip as a 2:2 complex. The two TEX12 chains (red) are bound to either end of an SYCE2 anti-parallel dimer (blue), with their C-termini oriented externally and immediately adjacent to SYCE2’s C-termini. The structure consists of a central four-helical bundle flanked by lateral three-helical bundles. (**b-c**) Schematics of the 2:2 crystal structure in which (b) the orientation of SYCE2 and TEX12 chains are highlighted, and (**c**) their additional S2C and Ctip sequences are shown as C-terminal coiled-coils. The 2:2 structure has a width of 2 nm and a length of 14 nm, which is predicted to increase to 19 nm upon addition of S2C and/or Ctip sequences. (**d**) The lateral three-helical bundle consists of an anti-parallel arrangement of SYCE2 chains and a single TEX12 chain, with a hydrophobic core formed by the residues indicated. (**e**) The transition point between lateral three-helical bundle and central four-helical bundle. (**f**) The central four-helical bundle consists of an anti-parallel arrangement of two TEX12 and two SYCE2 chains, with a hydrophobic core formed by the residues indicated. (**g-i**) SEC-SAXS analysis of SYCE2-TEX12 core ΔS2C/ΔCtip and ΔCtip. (**g**) SAXS scattering data overlaid with the theoretical scattering curves of the 2:2 crystal structure (red), with modelled C-terminal coiled-coil and S2C helix (blue), and with flexibly modelled N-termini (yellow); χ^2^ values are indicated and residuals for each fit are shown (inset). (**h**) SAXS *P(r)* interatomic distance distributions of SYCE2-TEX12 core ΔS2C/ΔCtip and ΔCtip, showing maximum dimensions (*Dmax*) of 15 nm and 19 nm, respectively. Their cross-sectional radii (Rc) were determined as 1.2 nm. (**i**) SAXS *ab initio* model of SYCE2-TEX12 core ΔS2C/ΔCtip. A filtered averaged model from 30 independent DAMMIF runs is shown with the 2:2 crystal structure docked into the SAXS envelope.

The enhanced stability of the SYCE2-TEX12 ΔCtip core complex facilitated the growth of protein crystals, which diffracted anisotropically to 2.42–3.36 Å resolution and enabled structure solution by molecular replacement of ideal helical fragments using *ARCIMBOLDO*40 (Table 1 and Extended Data Fig. 4a). This revealed a 2:2 helical assembly in which an anti-parallel SYCE2 dimer spans the 14-nm molecular length and positions TEX12 chains in a staggered configuration (Fig. 2a-c). TEX12 chains are arranged with their N-termini towards the midline and their C-termini at either end of the molecule, immediately adjacent to SYCE2’s N- and C-termini (Fig. 2a-c). The resultant 2:2 molecule has an unusual architecture in which a central four-helical bundle, formed of the SYCE2 dimer and overlapping N-terminal TEX12 chains, is flanked by three-helical bundles, each formed of the SYCE2 dimer and a single C-terminal TEX12 chain (Fig. 2a,d-f). Importantly, the C-terminal ends of SYCE2’s core were not visible in electron density. Thus, deletion of TEX12’s C-terminal tip corresponded with disorder of an analogous C-terminal sequence of the SYCE2 core (herein referred to as S2C; amino acids 155-165), such that the molecular ends of the 2:2 complex are formed of closely associated truncated C-termini of SYCE2 and TEX12 (Fig. 2a,c).

Does the 2:2 crystal structure represent the solution state of SYCE2-TEX12 ΔCtip? Size-exclusion chromatography small-angle X-ray scattering (SEC-SAXS) of the ΔCtip core 2:2 complex revealed a real-space *P(r)* interatomic distance distribution and Guinier analysis indicative of a rod-like molecule with 19-nm length and 1.2-nm cross-sectional radius (Fig. 2g,h, Extended Data Fig. 4b,c and Supplementary Table 1). These dimensions match the length of the crystal structure with missing S2C ends modelled as ideal helices, and its observed 2-nm width (Fig. 2c and Extended Data Fig. 4e). Further, the SAXS scattering curve was closely fitted by the crystal structure with modelled S2C helices and flexible N-termini (χ^2^ = 1.99; Fig. 2g and Extended Data Fig. 4e). We also analysed a ΔS2C/ΔCtip complex in which the C-termini of SYCE2 and TEX12 were deleted. SEC-MALS confirmed that the ΔS2C/ΔCtip core complex is predominantly 2:2 in solution (Extended Data Fig. 4d). SEC-SAXS determined a rod-like molecule with 15-nm length and 1.2-nm cross-sectional radius, with an *ab initio* envelope that matches the dimensions of the 2:2 crystal structure (Fig. 2g-i, Supplementary Table 1 and Extended Data Fig. 4c). Further, the SAXS scattering curve was closely fitted by the crystal structure upon ideal helical modelling of the few missing C-terminal amino-acids (χ^2^ = 1.23; Fig. 2g). We conclude that the 2:2 crystal structure corresponds to the solution state of the ΔCtip core complex and hence represents the obligate 2:2 structure of SYCE2-TEX12 that acts as its building-block for assembly.

### Crystal structure of SYCE2-TEX12 in a 4:4 assembly

**Extended Data Fig. 5:**
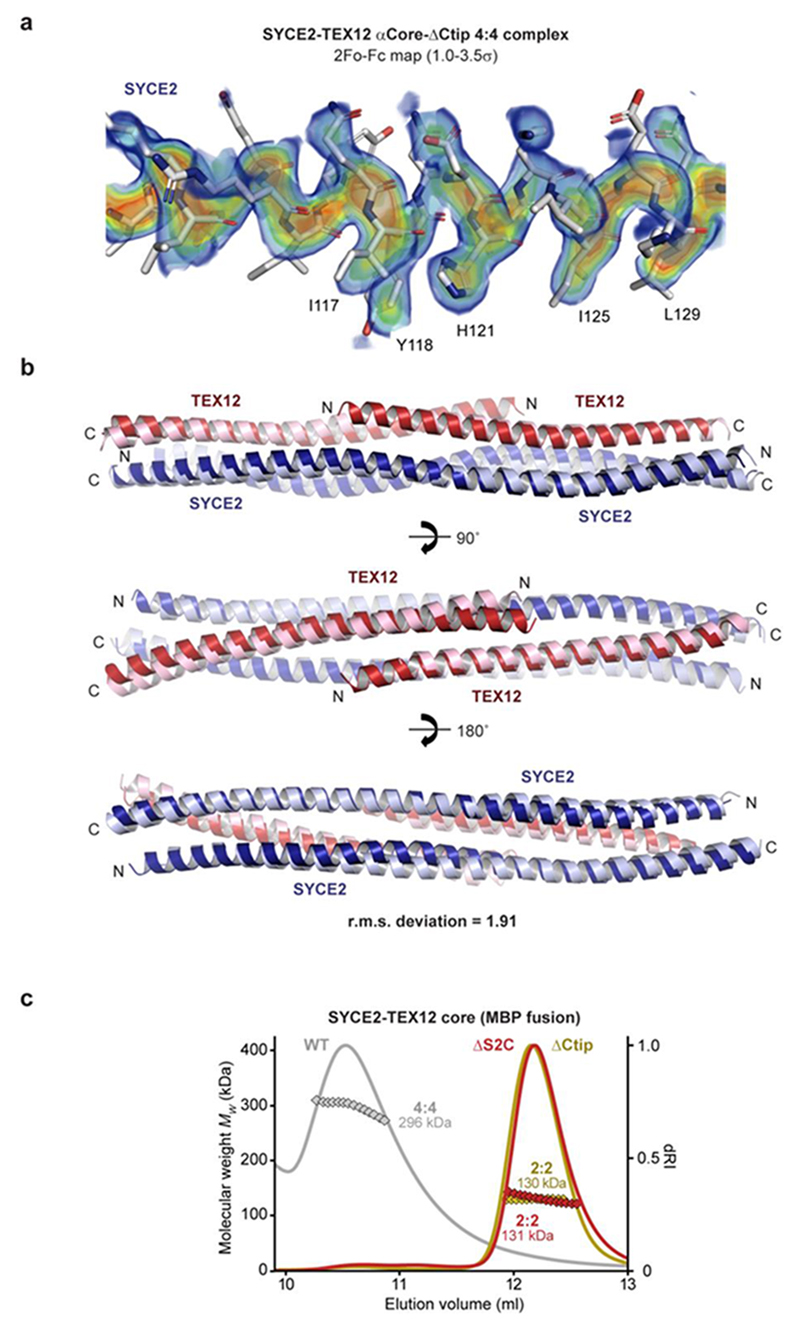
Crystal structure of the SYCE2-TEX12 core 4:4 complex. (**a**) 2Fo-Fc electron density map of the SYCE2-TEX12 core ΔCtip 4:4 complex (1.0σ), presented as a rainbow between 1.0σ (blue) and 3.5σ (red), superimposed on the refined crystallographic model. (**b**) Superposition of a constituent 2:2 complex from the SYCE2-TEX12 core ΔCtip 4:4 structure (light blue and light red) and the SYCE2-TEX12 core ΔCtip 2:2 structure (dark blue and dark red), with r.m.s. deviation of 1.91. (**c**) SEC-MALS analysis of SYCE2-TEX12 core MBP-fusion proteins (dRI profiles with molecular weights plotted as diamonds). SYCE2-TEX12 core wild-type is a 296 kDa 4:4 complex (theoretical – 278 kDa), whereas ΔS2C and ΔCtip form 131 kDa and 130 kDa 2:2 complexes, respectively (theoretical – 136 kDa and 137 kDa).

**Fig. 3:**
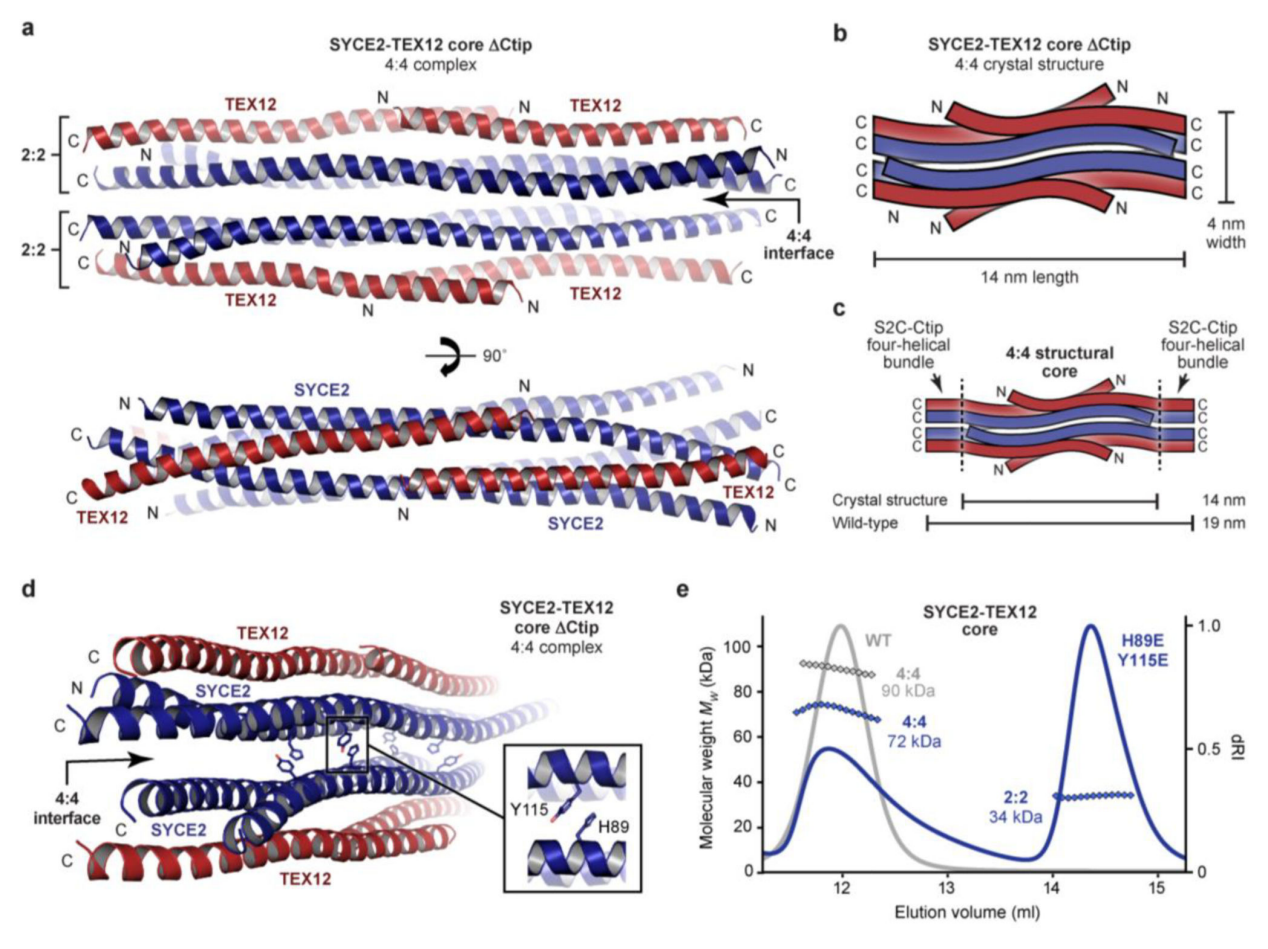
Crystal structure of the SYCE2-TEX12 core 4:4 complex. (**a**) Crystal structure of SYCE2-TEX12 core ΔCtip as a 4:4 complex. The structure consists of two 2:2 complexes interacting side-by-side through tessellation of their undulating SYCE2 surfaces at a 4:4 interface. This results in a central core of four SYCE2 chains (blue) flanked by two TEX12 chains (red) on either side. (**b-c**) Schematics of the 4:4 crystal structure in which (**b**) the orientation of SYCE2 and TEX12 chains are highlighted, and (**c**) their additional S2C and Ctip sequences are shown as C-terminal four-helical bundles. The 4:4 structure has a width of 4 nm and a length of 14 nm, which is predicted to increase to 19 nm upon addition of S2C and Ctip sequences. (**d**) Crystal structure of the SYCE2-TEX12 core ΔCtip 4:4 complex, highlighting interactions between SYCE2 residues H89 and Y115 at the 4:4 interface between juxtaposed 2:2 complexes. (**e**) SEC-MALS analysis of SYCE2-TEX12 core H89E/Y115E (dRI profiles with molecular weights plotted as diamonds) showing the formation of a 34 kDa 2:2 complex (60%; theoretical – 44 kDa) and 72 kDa 4:4 complex (40%; theoretical – 89 kDa). SYCE2-TEX12 core wild-type is shown in grey for comparison.

We obtained an alternative crystal form of SYCE2-TEX12 ΔCtip core, which diffracted anisotropically to 3.33–6.00 Å resolution, and was solved by molecular replacement using a three-helical bundle of the 2:2 structure as a search model (Table 1 and Extended Data Fig. 5a). This revealed a 4:4 structure in which two 2:2 complexes interact laterally, through tessellation of S-shaped SYCE2 dimers, to create a molecule of the same length (14 nm) but twice as wide (4 nm) as individual 2:2 complexes (Fig. 3a,b). The 2:2 crystal structure is largely retained by the two interacting 2:2 complexes (r.m.s. deviation = 1.91; Extended Data Fig. 5b), which seamlessly generate an SYCE2 tetrameric core with staggered TEX12 chains arranged on opposing surfaces (Fig. 3a). In common with the 2:2 structure, S2C sequences were missing from electron density, meaning that pairs of truncated SYCE2 and TEX12 C-termini are juxtaposed at both ends of the 4:4 molecule (Fig. 3c). The observed tessellation of 2:2 complexes provides an enticing mechanism for assembly of a 4:4 complex from structurally intact obligate 2:2 molecules, in agreement with our biochemical findings.

### TEX12’s Ctip stabilises the SYCE2-TEX12 4:4 complex

**Extended Data Fig. 6:**
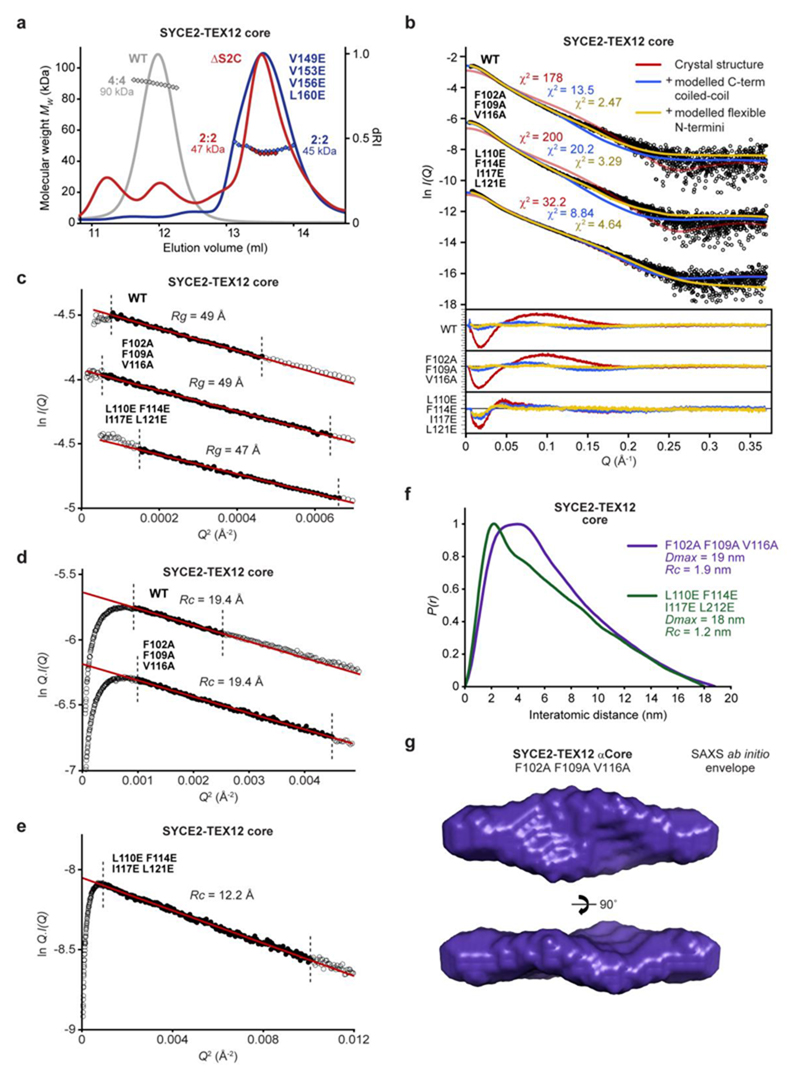
MALS and SAXS analysis of SYCE2-TEX12 wild-type and mutant complexes. (**a**) SEC-MALS analysis of SYCE2-TEX12 core ΔS2C and V149E, V153E, V156E and L160E mutants (dRI profiles with molecular weights plotted as diamonds), demonstrating the formation of 47 kDa and 45 kDa 2:2 complexes, respectively (theoretical - 42 kDa and 45 kDa). The wild-type 90 kDa 4:4 complex is shown in grey for comparison. (**b-g**) SEC-SAXS analysis of SYCE2-TEX12 core wild-type, F102A F109A V116A (FFV) and L110E F114E I117E L121E (LFIL) mutants. (**b**) SAXS scattering data overlaid with the theoretical scattering curves of the 4:4 crystal structure (red), with modelled Ctip-S2C C-terminal bundles (blue), and with flexibly modelled N-termini (yellow); χ^2^ values are indicated and residuals for each fit are shown (inset). (**c**) SAXS Guinier analysis to determine the radius of gyration (*Rg*); linear fits are shown in red, with the fitted data range highlighted in black and demarcated by dashed lines. The *Q.Rg* values were < 1.3 and *Rg* was calculated as 49 Å, 49 Å and 47 Å, respectively. (**d-e**) SAXS Guinier analysis to determine the radius of gyration of the cross-section (*Rc*); linear fits are shown in red, with the fitted data range highlighted in black and demarcated by dashed lines. The *Q.Rc* values were < 1.3 and *Rc* was calculated as (**d**) 19.4 Å for wild-type and FFV and (**e**) 12.2 Å for LFIL. (**f**) SAXS *P(r)* interatomic distance distributions of SYCE2-TEX12 core FFV and LFIL, showing maximum dimensions of 19 nm and 18 nm, respectively. (**g**) SAXS *ab initio* model of SYCE2-TEX12 core FFV in which a filtered averaged model from 30 independent DAMMIF runs is shown.

**Fig. 4:**
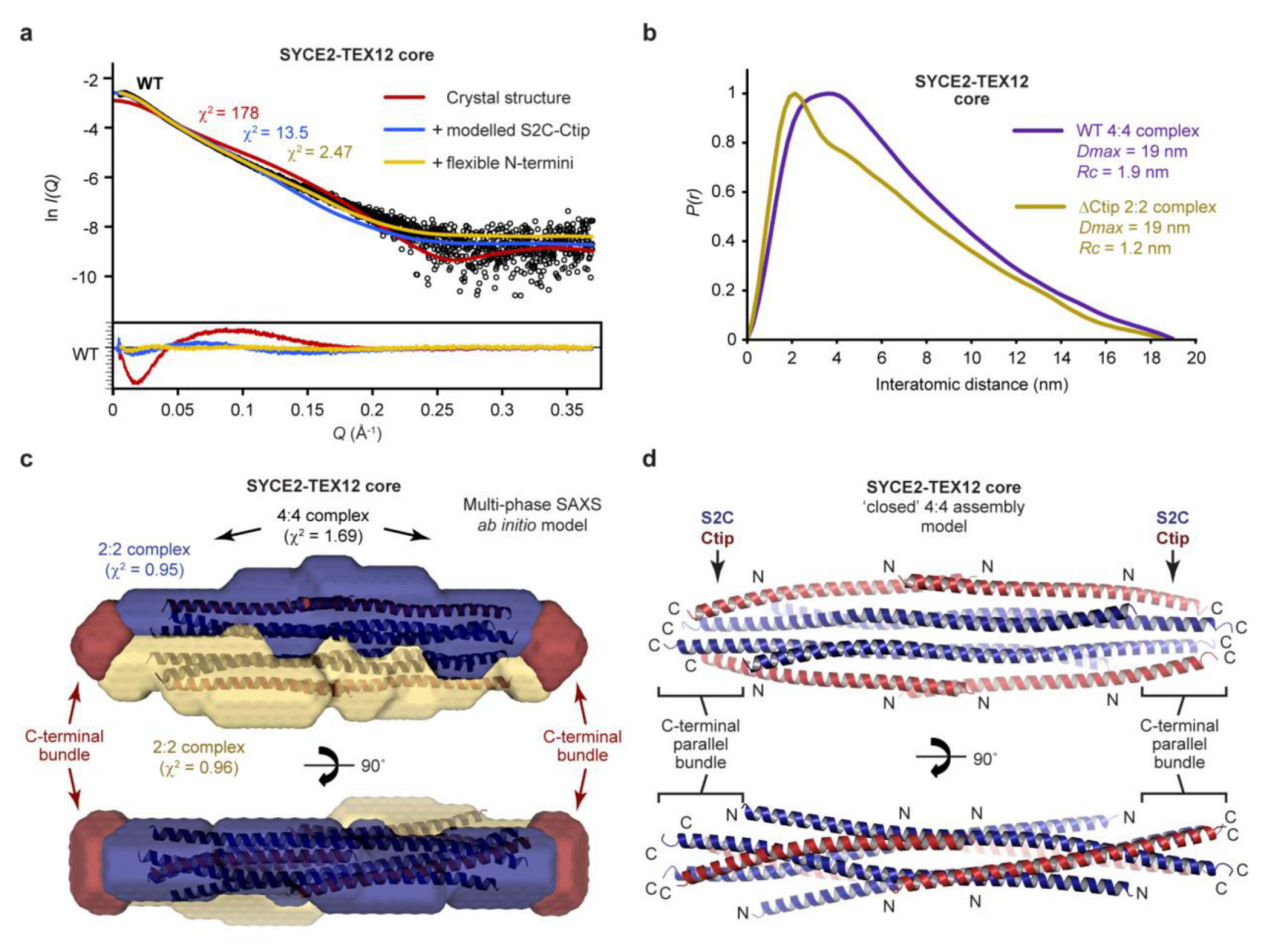
A molecular model of the SYCE2-TEX12 core ‘closed’ 4:4 assembly. (**a-c**) SEC-SAXS analysis of SYCE2-TEX12 core. (**a**) SAXS scattering data of SYCE2-TEX12 core overlaid with the theoretical scattering curves of the 4:4 crystal structure (red), with modelled Ctip-S2C C-terminal bundles (blue), and with flexibly modelled N-termini (yellow); χ^2^ values are indicated and residuals for each fit are shown (inset). (**b**) SAXS *P(r)* interatomic distance distributions of SYCE2-TEX12 core wild-type and ΔCtip, showing maximum dimensions (*Dmax*) of 19 nm. Their cross-sectional radii (*Rc*) were determined as 1.9 nm and 1.2 nm, respectively. (**c**) Multi-phase SAXS *ab initio* (MONSA) model of the SYCE2-TEX12 core 4:4 complex (entire envelope; χ^2^=1.69), consisting of two SYCE2-TEX12 core ΔS2C/ΔCtip complexes (blue and yellow envelopes; χ^2^=0.95 and 0.96) and remaining mass corresponding to S2C-Ctip C-terminal bundles (red). (**d**) Theoretical model of the SYCE2-TEX12 core ‘closed’ 4:4 complex in which S2C-Ctip hetero-dimeric coiled-coil models were docked onto the 4:4 crystal structure and assembled into C-terminal four-helical bundles through iterative energy minimisation and geometry idealisation.

The 4:4 crystal structure lacks TEX12’s C-terminal tip, which is required for 4:4 assembly in solution, suggesting that this conformation was supported by the crystal lattice. We reasoned that the tessellating interface likely drives 4:4 assembly of the wild-type protein and is stabilised in solution by additional TEX12 Ctip interactions. To test this, we introduced glutamate mutations of amino-acids H89 and Y115, which mediate the tessellating interaction but have no structural role within constituent 2:2 complexes (Fig. 3d). Within the core complex, the H89E Y115E mutation partially blocked 4:4 assembly, restricting 60% of material to a 2:2 complex, despite the presence of Ctip sequences (Fig. 3e). SEC-SAXS determined that the wild-type 4:4 complex has a 19-nm length and 1.9-nm cross-sectional radius (Fig. 4a,b and Extended Data Fig. 6b-d), matching the observed geometry of the 4:4 crystal structure in which the length of the 2:2 complex is retained and its width is doubled from 2 nm to 4 nm (Fig. 3a-c). Further, multi-phase SAXS *ab initio* modelling of the wild-type 4:4 structure placed ΔCtip 2:2 *ab initio* envelopes in parallel, consistent with their lateral tessellating interaction, with missing mass – corresponding to S2C and Ctip sequences – placed at either end of the molecule (χ^2^ = 1.69; Fig. 4c). Thus, the lateral tessellating interaction of 2:2 complexes within the 4:4 crystal structure describes the underlying architecture of the wild-type 4:4 assembly.

**Extended Data Fig. 7:**
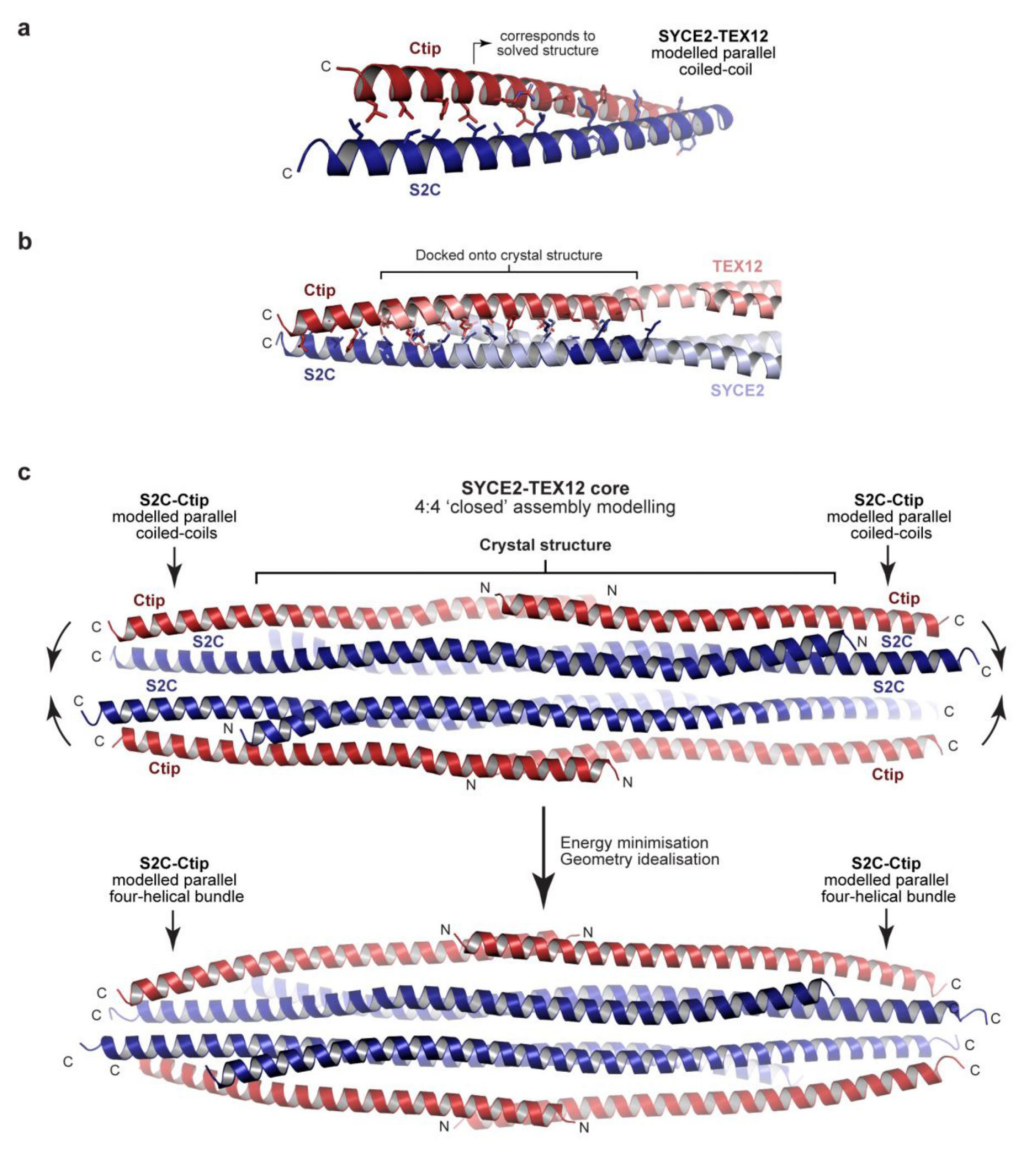
Modelling of the SYCE2-TEX12 core ‘closed’ 4:4 assembly. (**a-c**) Modelling of the ‘closed’ 4:4 assembly. (**a**) Theoretical model of hetero-dimeric coiled-coils corresponding to SYCE2 and TEX12 amino-acids 114-165 and 75-123, respectively (including Ctip and S2C sequences 155-165 and 114-123), were generated using *CCBuilder* by specifying the heptad register observed in the 2:2 and 4:4 crystal structures. (**b**) The coiled-coil model (red and blue) docked onto the 2:2 ends of the 4:4 crystal structure (pale red and pale blue), showing close correspondence between overlapping helices. (**c**) This resulted in a 4:4 structure with emanating S2C-Ctip dimers (top) that was subjected to iterative energy minimisation and geometry idealisation such that S2C-Ctip sequences of adjacent dimers combined into capping four-helical bundles at either end of the 4:4 molecule (bottom).

How does TEX12’s Ctip stabilise the wild-type 4:4 complex? The location of pairs of truncated TEX12 and SYCE2 C-termini at either end of the 4:4 complex suggests that Ctip and S2C sequences could interact in four-helical structures that clamp together tessellated 2:2 molecules. Accordingly, deletion of SYCE2’s S2C blocked 4:4 assembly and restricted the SYCE2-TEX12 core to a 2:2 complex (Extended Data Fig. 6a), confirming that S2C and Ctip have equivalent roles in assembly. The predicted heptads of S2C-Ctip sequences are in-phase with the crystallographically-observed heptads of their upstream sequences, so we modelled an extended S2C-Ctip parallel heterodimeric coiled-coil (Extended Data Fig. 7a). This model docked seamlessly and in-phase with overlapping sequences within the 4:4 crystal structure (Extended Data Fig. 7b), producing a model in which S2C-Ctip coiled-coils emanate from both ends of constituent 2:2 complexes (Extended Data Fig. 7c). Upon energy minimisation and geometry idealisation, juxtaposed pairs of S2C-Ctip dimers interacted together (Extended Data Fig. 7c), forming S2C-Ctip 2:2 parallel bundles that clamp together 2:2 complexes at either end of the tessellating 4:4 interface (Fig. 4d). The resultant ‘clamped’ 4:4 structure closely fitted the SAXS scattering curve of the wild-type 4:4 core complex upon flexible modelling of missing N-termini (χ^2^ = 2.47; Fig. 4a). Thus, formation of S2C-Ctip C-terminal bundles provides a mechanism for clamping together laterally associated 2:2 complexes within the architecture of the wild-type 4:4 complex.

### Controlling the hierarchical assembly of SYCE2-TEX12

**Fig. 5:**
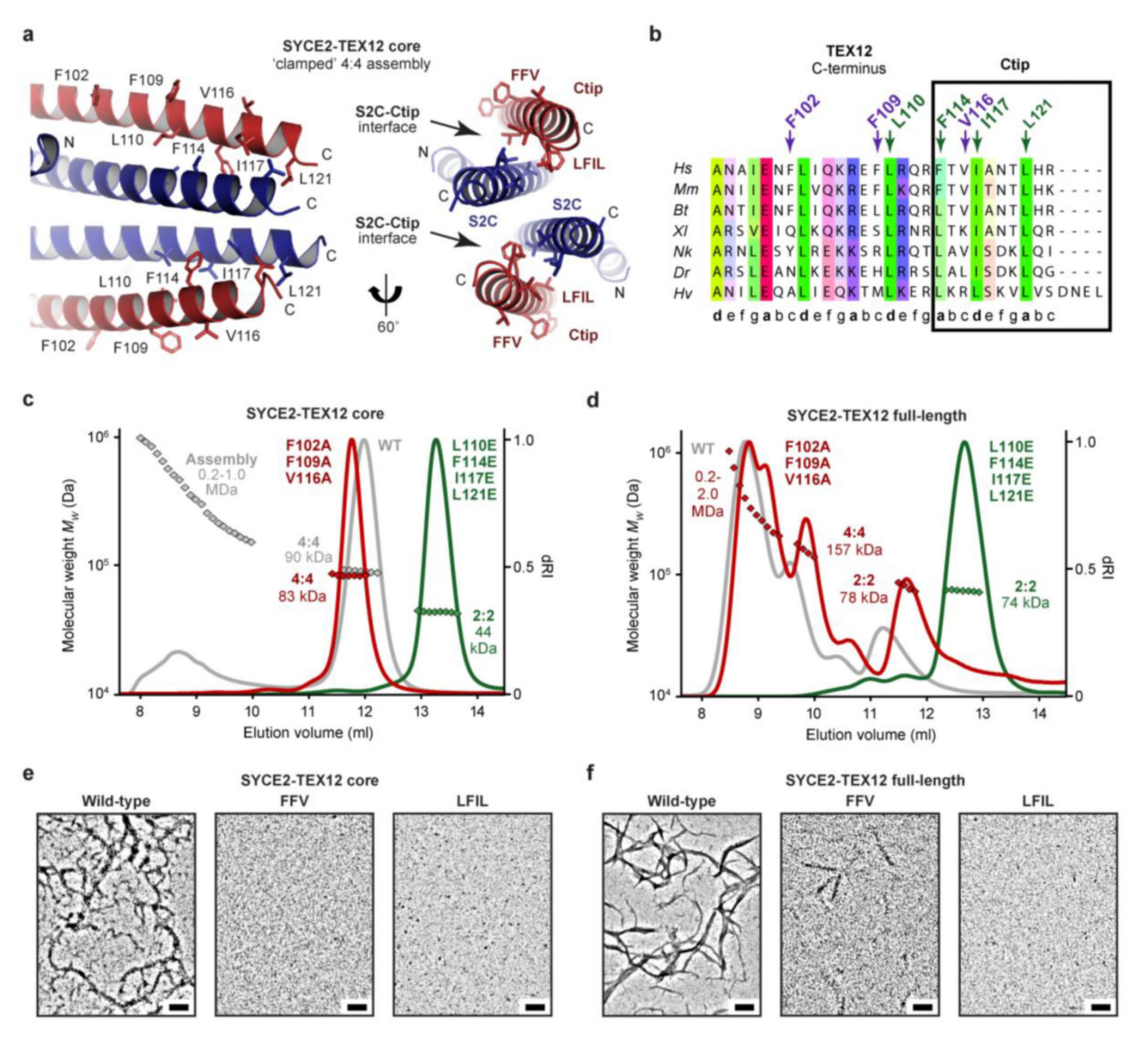
Molecular determinants of SYCE2-TEX12 self-assembly by TEX12’s C-terminal tip. (**a**) Molecular model of the SYCE2-TEX12 core ΔCtip ‘closed’ 4:4 assembly. The C-terminal bundle is formed of interactions between two S2C-Ctip coiled-coils, each of which involves heptad interactions between S2C residues V149, V153, V156 and L160, and Ctip residues L110, F114, I117 and L121, whilst F102, F109 and V116 are solvent-exposed. (**b**) Multiple sequence alignment of the TEX12 C-terminus, highlighting the Ctip sequence and the presence of LFIL residues at heptad positions, and FFV residues at non-heptad positions. (**c-d**) SEC-MALS analysis of SYCE2-TEX12 (**c**) core and (**d**) full-length mutants F102A F109A V116A (FFV, red) and L110E F114E I117E L121E (LFIL, green), with wild-type shown in grey for comparison (dRI profiles with molecular weights plotted as diamonds). (**c**) SYCE2-TEX12 core FFV forms an 83 kDa 4:4 complex (theoretical – 88 kDa) with no higher-order assemblies, whilst LFIL forms a 44 kDa 2:2 complex (theoretical – 45 kDa). (**d**) SYCE2-TEX12 full-length FFV forms a 78 kDa 2:2 complex (15%; theoretical – 78 kDa), 155 kDa 4:4 complex (20%; theoretical – 156 kDa) and large molecular assemblies of up to 2.0 MDa (65%), whilst LFIL forms a 74 kDa 2:2 complex (theoretical – 78 kDa). (**e,f**) Electron microscopy of SYCE2-TEX12 (**e**) core and (**f**) full-length mutants. FFV inhibits SYCE2-TEX12 core assembly and restricts full-length to occasional fibres. LFIL inhibits SYCE2-TEX12 core and full-length fibrous assembly. Representative of at least three independent experiments. Scale bars, 100 nm.

Our model of the clamped 4:4 assembly predicts that TEX12 amino-acids L110, F114, I117 and L121 contribute to the C-terminal bundle’s hydrophobic core, whilst F102, F109 and V116 are solvent-exposed (Fig. 5a,b). Thus, the first group is likely essential for clamped 4:4 assembly, whereas the second group is likely dispensable. Accordingly, hydrophobic core mutation L110E F114E I117E L121E (LFIL) restricted core and full-length SYCE2-TEX12 to 2:2 complexes and blocked fibrous assembly, thereby mimicking ΔCtip (Fig. 5c-f). We similarly observed a restricted 2:2 complex upon analogous mutation of S2C hydrophobic core amino-acids V149, V153, V156 and L160 (Fig. 5a and Extended Data Fig. 6a). In contrast, surface mutation F102A F109A V116A (FFV) retained the 4:4 core complex, and wild-type pattern of full-length assembly in solution, but blocked higher-order assembly (Fig. 5c-f and Extended Data Fig. 3b,d). Importantly, SEC-SAXS confirmed that LFIL and FFV mutants adopt native 2:2 and 4:4 core structures, respectively (Extended Data Fig. 6b-g). Hence, whilst the core complex relies solely on FFV amino-acids for assembly, the full complex undergoes residual assembly through its unstructured termini, with FFV residues required for full fibre formation. Thus, we have identified separation-of-function mutations that block SYCE2-TEX12 in its building-block 2:2 and intermediate assembly structures. The mimicry of ΔCtip by LFIL mutation validates our clamped 4:4 assembly model, whilst blockade of higher-order assembly by FFV mutation implicates these surface-exposed residues in SYCE2-TEX12 fibre assembly.

### SYCE2-TEX12 forms 2-nm and 4-nm fibres

**Fig. 6:**
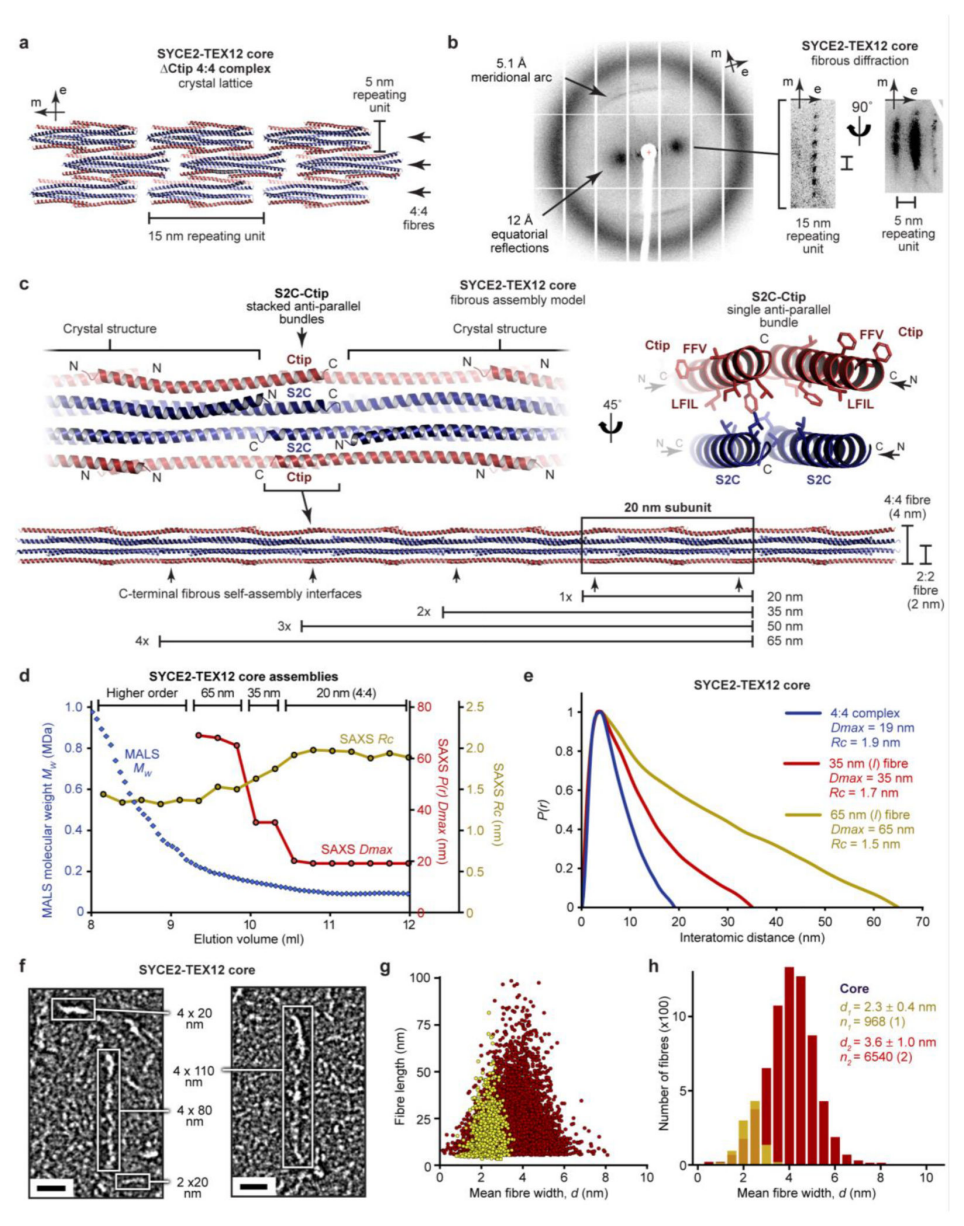
A molecular model of the SYCE2-TEX12 core fibrous assembly. (**a**) The SYCE2-TEX12 core ΔCtip 4:4 crystal lattice is formed of 15 nm translations along the meridional axis that generate 4:4 fibres, which associate laterally as 5 nm repeating units. (**b**) X-ray diffraction pattern of SYCE2-TEX12 core ΔCtip crystals demonstrating 5.1 Å meridional arcs and 12 Å equatorial reflections, which resolve upon rotation about the meridional axis into longitudinal and lateral spacings corresponding to 15 nm and 5 nm repeating units, respectively. (**c**) Theoretical model of SYCE2-TEX12 core fibrous assembly in which adjacent 4:4 complexes are translated by 15 nm and interact back-to-back through stacked S2C-Ctip anti-parallel four-helical bundles (left), of which each bundle contains a hydrophobic core of Ctip LFIL and S2C residues, with FFV residues remaining solvent-exposed (right). This assembly mechanism generates fibres of discrete lengths corresponding to the 20 nm initial subunit and integer multiples of the 15 nm repeating unit, with a width of 2 nm or 4 nm if it is formed of 2:2 or 4:4 complexes, respectively. (**d-e**) SEC-SAXS analysis of SYCE2-TEX12 core assemblies. (**d**) SAXS *P(r)* maximum dimensions (*Dmax,* red), cross-sectional radii (*Rc*, yellow) and corresponding MALS molecular weights (diamonds, blue) are shown across an elution peak; *Dmax* is not determined for higher-order species in which the Shannon limit is exceeded. (**e**) SAXS *P(r)* interatomic distance distributions of the SYCE2-TEX12 core 4:4 complex (blue) and its fibrous assemblies of lengths 35 nm (red) and 65 nm (yellow). (**f-h**) Electron microscopy of SYCE2-TEX12 core in which (**f**) electron micrographs reveal molecules of 2 x 20 nm, 4 x 20 nm, 4 x 80 nm and 4 x 110 nm (w x l). Scale bars, 20 nm. (**g**) Scatter plots of mean fibre width (*d,* nm) against fibre length (nm), and (**h**) histograms of the number of fibres with mean widths within 0.5-nm bins, for two distinct populations (2 nm, yellow; 4 nm, red). The mean, standard deviation and number of fibres in each population were determined from one and two micrographs, respectively.

How does TEX12’s Ctip drive assembly of SYCE2-TEX12 fibres? The crystal lattice of the 4:4 structure is a fibrous array in which 4:4 molecules are arranged in end-to-end chains, with 1-nm gaps between juxtaposed C-termini, and a lateral stagger (Fig. 6a). Could this represent the fibrous structure of SYCE2-TEX12, with end-to-end chains corresponding to fibres of 4:4 molecules that are thickened by lateral staggering? We obtained protein crystals of various SYCE2-TEX12 core constructs, including ΔCtip, which displayed a fibrous pattern of X-ray diffraction that is typical of the k-m-e-f family (keratin, myosin, epidermin, fibrinogen) of fibrous α-proteins (Fig. 6b)^41,42^. This consists of a 5.1 Å meridional arc arising from the coiled-coil repeat, and 12 Å equatorial reflections that correspond to inter-helical distances of three-/four-helical bundles (contrasting with 9.8 Å for dimeric coiled-coils)^41,42^. This is consistent with the end-to-end arrangement of molecules within the 4:4 crystal lattice. Further, upon rotation about the meridional axis, equatorial reflections resolved into orthogonal 15-nm and 5-nm regularly-spaced reflections, closely matching the repeating units of the 4:4 crystal lattice (Fig. 6a,b). Thus, the fibrous diffraction pattern is consistent with the fibrous array of the 4:4 crystal lattice, demonstrating that this molecular arrangement is commonly exhibited by SYCE2-TEX12 constructs.

**Extended Data Fig. 8:**
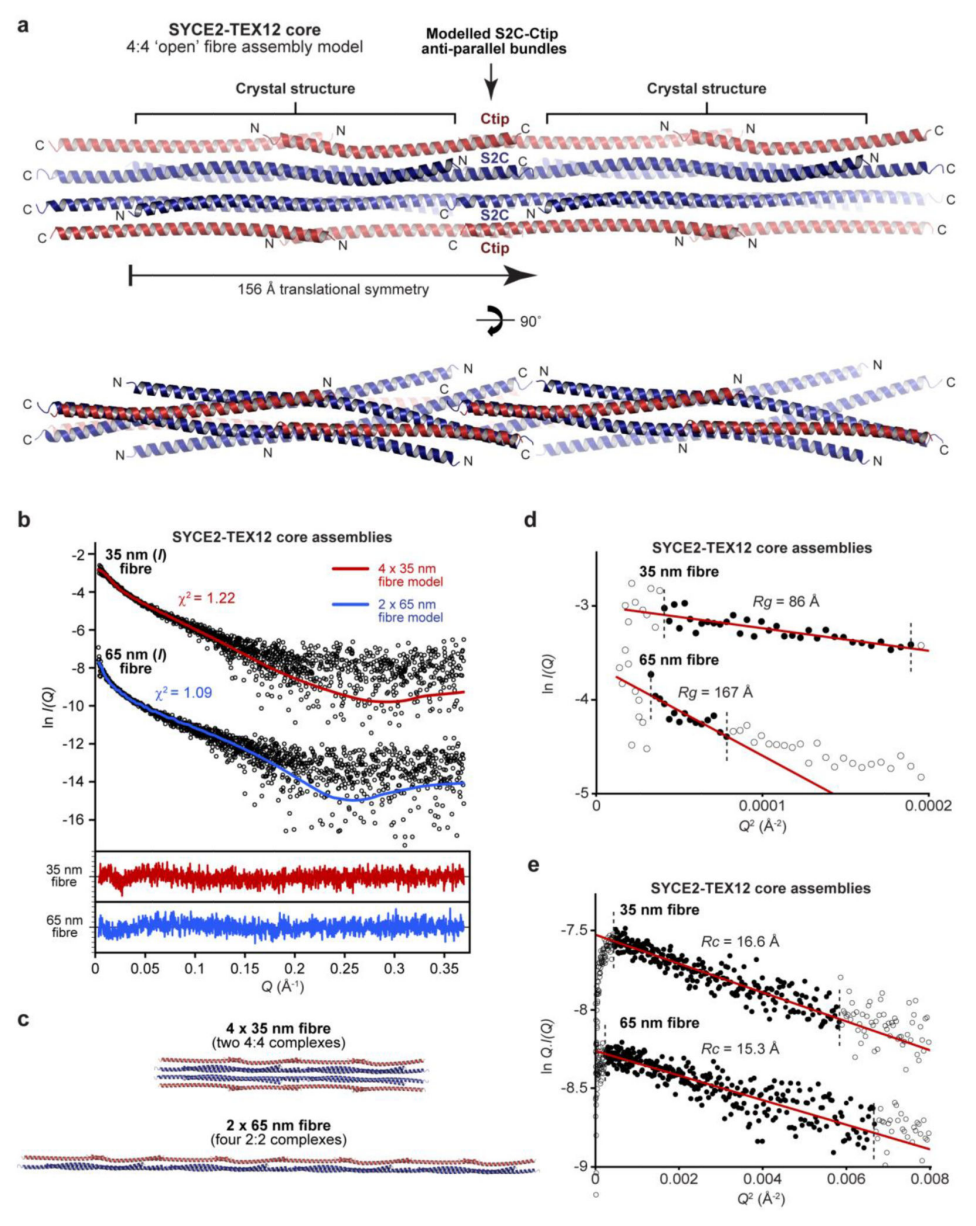
Modelling of the SYCE2-TEX12 core fibrous assembly. (**a**) Model of SYCE2-TEX12 core fibrous assembly in which adjacent 4:4 complexes are translated by 15 nm and interact back-to-back through stacked S2C-Ctip anti-parallel four- helical bundles. (**b-e**) SEC-SAXS analysis of SYCE2-TEX12 core 35 nm and 65 nm (length) fibres. (**b**) SAXS scattering data overlaid with the theoretical scattering curves of a 4 x 35 nm (w x l) fibre model (two consecutive 4:4 complexes, red) and a 2 x 65 nm (w x l) fibre model (four consecutive 2:2 complexes, blue); χ^2^ values are indicated and residuals for each fit are shown (inset). (**c**) The 4 x 35 nm and 2 x 65 nm fibre models used for SAXS data fitting. (**d**) SAXS Guinier analysis to determine the radius of gyration (*Rg*); linear fits are shown in red, with the fitted data range highlighted in black and demarcated by dashed lines. The *Q.Rg* values were < 1.3 and *Rg* was calculated as 86 Å and 167 Å, respectively. (**e**) SAXS Guinier analysis to determine the radius of gyration of the cross-section (*Rc*); linear fits are shown in red, with the fitted data range highlighted in black and demarcated by dashed lines. The *Q.Rc* values were < 1.3 and *Rc* was calculated as 16.6 Å and 15.3 Å, respectively.

If the 4:4 crystal lattice represents the true fibrous assembly, then it should be possible to rationalise S2C-Ctip’s structural role within this fibrous array. We modelled S2C and Ctip as α-helical extensions of SYCE2 and TEX12 chains within 4:4 complexes *in situ.* The modelled S2C-Ctip helices of juxtaposed 2:2 ends immediately tessellated and interacted favourably within anti-parallel bundles, with two stacked S2C-Ctip bundles mediating end-to-end association of 4:4 molecules (Fig. 6c and Extended Data Fig. 8a). Importantly, LFIL and S2C heptad residues are in the hydrophobic core, whilst FFV residues are surface-exposed, in agreement with the clamped 4:4 model and their mutant phenotypes (Fig. 6c). Our molecular model suggests that constituent SYCE2-TEX12 fibres could be 2-nm or 4-nm wide, formed from 2:2 or 4:4 building-blocks, and stabilised by individual S2C-Ctip bundles or the tessellating 4:4 interface with stacked bundles (Fig. 6c). Further, it predicts that the lengths of constituent fibres are defined by a 20-nm initial subunit with 15-nm increments for additional subunits (Fig. 6c).

We tested the fibre assembly model through SAXS analysis across the size-exclusion chromatography elution profile of SYCE2-TEX12 core (Fig. 6d). There was a stepwise increase in molecular length from 19 nm (4:4) to 35 nm and 65 nm at elution points preceding the 4:4 complex, with a concomitant reduction in cross-sectional radius from 1.9 nm to 1.4 nm, suggesting a progression from pure 4:4 fibres (1.9-nm radius; 4-nm width) to a mixture with 2:2 fibres (1.2-nm radius; 2-nm width) (Fig. 6d,e and Supplementary Table 1). The 35 nm and 65 nm species correspond to the predicted lengths of end-to-end assemblies, and their SAXS scattering curves were fitted by modelled fibres of two 4:4 complexes (4 x 35 nm; χ^2^ = 1.22) and four 2:2 complexes (2 x 65 nm; χ^2^ = 1.09), respectively (Extended Data Fig. 8b-e and Supplementary Table 1). We confirmed these findings by visualising the smallest SYCE2-TEX12 core assemblies within negatively-stained regions of electron micrographs (Fig. 6f). Automated image analysis determined populations with mean fibre widths of 2.3 ± 0.4 nm and 3.6 ± 1.0 nm, corresponding to 2:2 (2-nm width) and 4:4 (4-nm width) fibres, respectively (Fig. 6f-h). Further, the observed lengths of 2-nm and 4-nm fibres conformed to the predicted discrete lengths of end-to-end assemblies (Fig. 6f). Thus, SAXS and EM data support our molecular model of SYCE2-TEX12 assembly into 2-nm and 4-nm fibres.

### Hierarchical assembly of SYCE2-TEX12 fibres

**Fig. 7:**
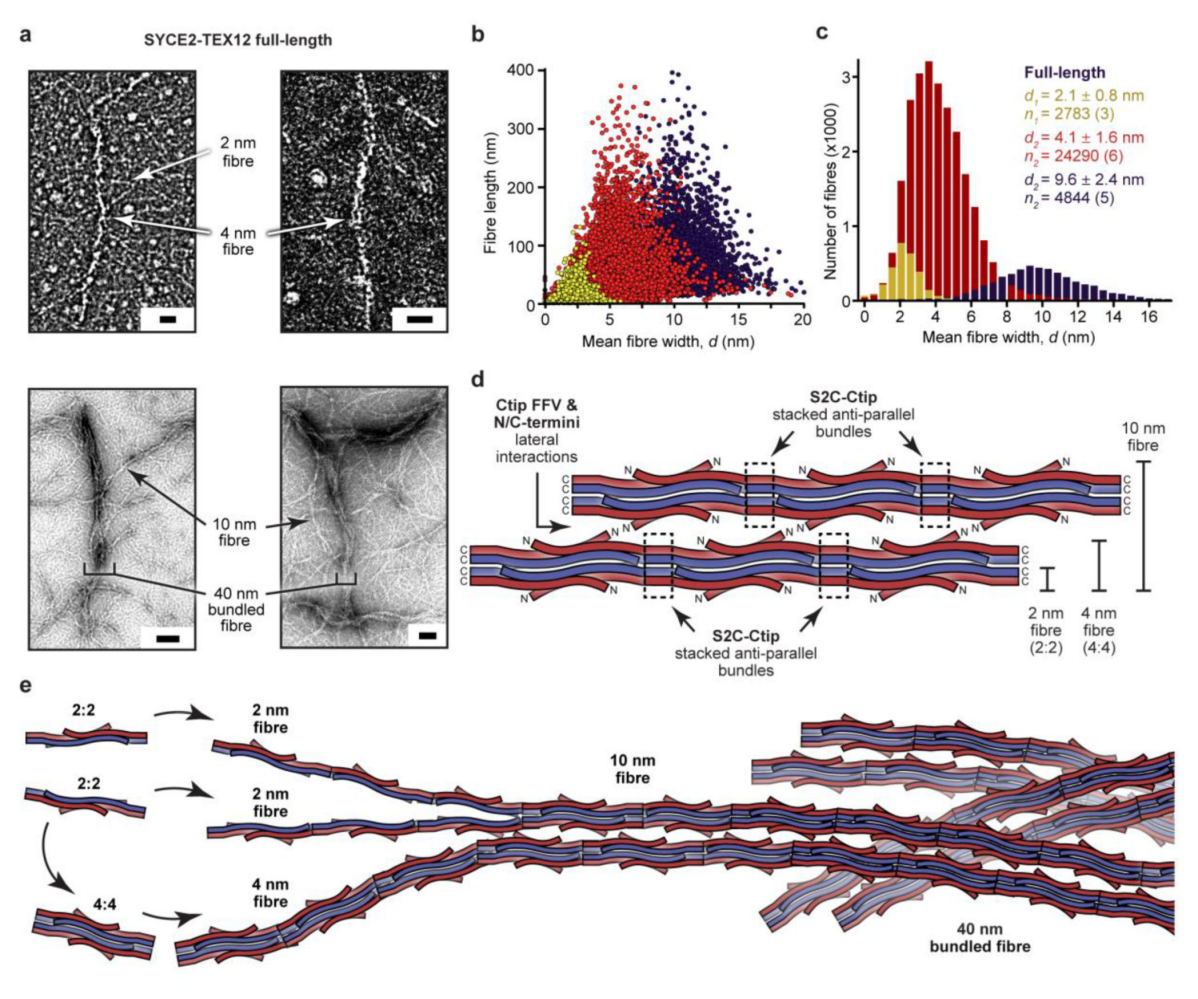
A molecular mechanism for fibrous assembly of SYCE2-TEX12. (**a-c**) Electron microscopy of full-length SYCE2-TEX12 in which (**a**) electron micrographs reveal the presence of 2 nm, 4 nm and 10 nm fibres, which become intertwined in bundled fibres of up to 40 nm in width. Scale bars, 20 nm (top) and 50 nm (bottom). (**b**) Scatter plots of mean fibre width (*d*, nm) against fibre length (nm), and (**c**) histograms of the number of fibres with mean widths within 0.5 nm bins, for three distinct populations (2 nm, yellow; 4 nm, red; 10 nm, blue). The mean, standard deviation and number of fibres in each population were determined from three, six and five micrographs, respectively. (**d**) Schematic of SYCE2-TEX12 fibrous assembly in which stacked S2C-Ctip anti-parallel bundles mediate end-to-end associations between subunits that generate 2-nm fibres and 4-nm fibres from 2:2 and 4:4 complexes, respectively. 10-nm fibres form through the lateral association of 4:4 fibres, mediated by Ctip FFV and N/C-terminal interactions. (**e**) Model of hierarchical fibrous assembly by SYCE2-TEX12. The building-block 2:2 complexes can assemble directly into 2-nm fibres, which then associate into 4-nm fibres, or can aggregate into ‘closed’ 4:4 structures that directly assemble into 4-nm fibres. The lateral association of 4-nm fibres generates 10-nm fibres, which become intertwined in bundled fibres of up to 40 nm width and many micrometres in length, and fulfil the known dimensions of the SC central element.

How do 2-nm and 4-nm fibres assemble into 40-nm fibres? We visualised full-length SYCE2-TEX12 within negatively-stained regions of electron micrographs (Fig. 7a). This revealed the presence of 2-nm (2.1 ± 0.8 nm) and 4-nm (4.1 ± 1.6 nm) fibres, corresponding to the 2:2 and 4:4 fibres observed for the core complex, but of considerably greater length (Fig. 7a-c). Importantly, the full-length complex also formed 10-nm fibres (9.6 ± 2.4 nm), which frequently intertwined within bundles of up to 40 nm that accumulated stain (Fig. 7a-c). The 10-nm fibres likely represent laterally-associated 4:4 fibres, interacting through Ctip/FFV residues and flanking termini, and seemingly represent an upper limit for the width of individual ‘smooth’ fibres (Fig. 7d,e). The 40-nm bundled fibres demonstrated no overt helical or linear structural regularity, but instead represent an irregular packing of 10-nm fibres, consistent with gliding surface interactions. The resultant fibres have the same dimensions as the positively-stained SYCE2-TEX12 fibres that dominate electron micrographs (Fig. 1f,g), suggesting that they could represent fortuitously stained representative structures, or could be a subpopulation of loosely-packed fibres. Further, whilst we lack information on the internal structure of the SC central element, its overall dimensions and polymorphic appearance match those of the 40-nm fibres (Fig. 1a), consistent with SYCE2-TEX12 constituting the major structural component of the central element.

In summary, SYCE2-TEX12 undergoes fibrous assembly through a hierarchical mechanism in which building-block 2:2 molecules laterally tessellate into 4:4 molecules, and assemble via end-to-end S2C-Ctip bundles into 2-nm and 4-nm fibres (Fig. 7d,e). These structures thicken into 10-nm fibres, which are interwoven within 40-nm bundled fibres (Fig. 7d,e). Hence, we define the molecular mechanism whereby SYCE2-TEX12 self-assembles into rope-like fibres that structurally underpin longitudinal growth of the SC to enable synaptic elongation along the axial length of meiotic chromosomes.

## Discussion

The formation of supramolecular cellular structures through protein self-assembly is fundamentally important in numerous physiological systems, including cytoskeletal and chromosomal structure, and pathological processes such as amyloid formation^43–45^. Self-assembling systems can be defined as obligate building blocks that interact recursively to generate assembled structures. In meiotic chromosome synapsis, the supramolecular SC is formed by three major self-assembling systems: a zipper-like SYCP1 lattice, chromosome-associated SYCP3 (and SYCP2-SYCP3) axes, and a midline SYCE2-TEX12 fibrous backbone^17,31,32,36^. Here, we report that SYCE2-TEX12 is formed of 2:2 hetero-tetrameric building blocks, which assemble into fibres through short motifs at their C-terminal α-helical ends. The deletion of TEX12’s C-terminal tip blocked fibrous assembly and enabled X-ray crystallographic structure solution of its 2:2 building-block complex, revealing an unusual three- and four-helical coiled-coil structure. SYCP1-3 consist of tetrameric rod-like building blocks that self-assemble via N- and C-terminal α-helical end motifs^17,32,36^. Thus, an apparent theme in mammalian SC assembly is the formation of tetrameric coiled-coil building blocks, which self-assemble via α-helical end motifs that may be disrupted to stabilise their non-assembled building-block states in solution.

The assembly motifs of SYCE2-TEX12 mediate self-assembly by forming parallel S2C-Ctip bundles that clamp together laterally-tessellated 2:2 molecules in discrete 4:4 structures, and anti-parallel S2C-Ctip bundles that provide end-to-end 2:2 and 4:4 molecular interactions within 2-nm and 4-nm fibres, respectively. These clamped and fibrous conformations are stabilised by heptad interactions of Ctip (LFIL) and S2C amino acids (Fig. 7d,e). Ctip’s surface-exposed (FFV) amino acids support the fibrous conformation by mediating thickening and intertwining into 10-nm ‘smooth’ fibres and mature 40-nm bundled fibres (Fig. 7d,e). There are clear parallels with SYCP1-3, in which supramolecular assembly is mediated by anti-parallel end-to-end interactions of bioriented SYCP1 molecules^17^, and of linearly-organised SYCP3/SYCP2-SYCP3 molecules^32,36,37^. However, their resultant assemblies are structurally and functionally distinct owing to subtle differences in how these common principles are implemented.

Full-length SYCE2-TEX12 shows a higher propensity for assembly than its structural core, and exists in a far wider range of solution species, including 2:2, 4:4 and megadalton complexes. Thus, SYCE2 and TEX12 unstructured termini likely provide additional longitudinal stability to cores assemblies formed through lateral and end-to-end interfaces. In their absence, a compensatory increase in the number of end-to-end interfaces within the cross-section, through fibre thickening, may be necessary for stability, explaining our EM finding that core fibres are slightly thicker than full-length fibres. Further, unstructured termini could stabilise 2:2 complexes by acting in ‘cis’ through termini interacting with their own core, explaining the formation of wild-type 2:2 complexes by full-length but not core SYCE2-TEX12. Thus, the core complex may represent a restricted form of SYCE2-TEX12 in which the absence of unstructured termini reduces the stability of both higher-order assemblies and building-block 2:2 complexes, resulting in its stabilisation within a partially-assembled clamped 4:4 structure.

How does the SYCE2-TEX12 structure relate to our previous findings that isolated SYCE2 and TEX12 form tetramers and dimers, respectively^31^? The SYCE2 tetramer interface within the 4:4 structure is disrupted by TEX12 ΔCtip so is unlikely to support an isolated tetramer, and the linear arrangement of TEX12 chains is incompatible with isolated TEX12’s compact dimer structure^46^. Thus, these likely represent alternative conformations. Isolated SYCE2 is unstable in solution and has no known cellular role, so the tetramer may be artefactual^31^. In contrast, isolated TEX12 is highly stable and has a defined cellular function in centrosomes, so likely represents a physiological conformation^31,46^. These findings reflect a wider phenomenon that coiled-coils often interact through conformational change in which homotypic interfaces are replaced by heterotypic interfaces, rather than through additive binding of existing oligomers. Thus, whilst we previously attributed the 4:4 complex to an SYCE2 tetramer binding to two TEX12 dimers^31^, it is now apparent that these species undergo conformational change into a structure that consists of interfaces distinct from those of its isolated protein constituents.

**Extended Data Fig. 9:**
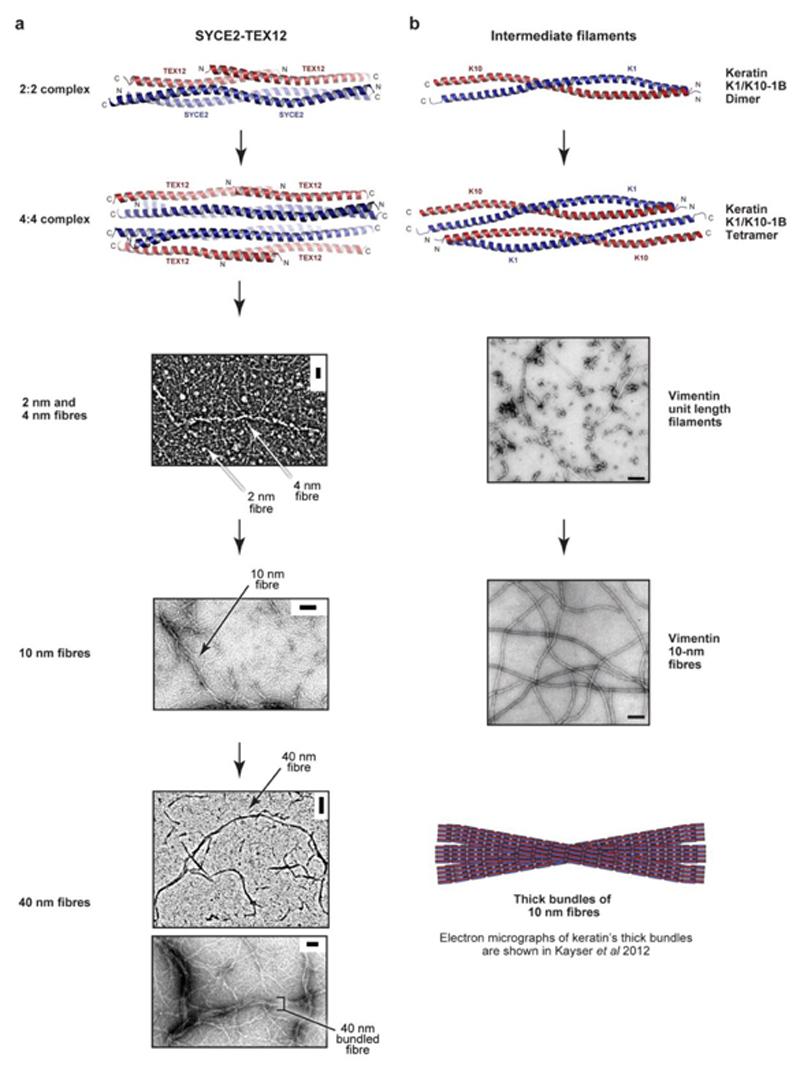
Comparison of hierarchical assembly by SYCE2-TEX12 and intermediate filaments. (**a,b**) Side-by-side comparison of assembly by (**a**) SYCE2-TEX12 and (**b**) intermediate filaments, using examples of keratin and vimentin. (**a**) SYCE2-TEX12 undergoes hierarchical assembly from a 2:2 into4:4 complex, and through end-on assembly into 2-nm and 4-nm fibres, which assemble into 10-nm fibres that become bundled together in 40-nm fibres that represent its dominant state by electron microscopy. Scale bars, 20 nm (top), 50 nm (middle), 100 nm (bottom, upper) and 50 nm (bottom, lower). (**b**) The keratin K1/K10-1B complex is a dimer that forms an anti-parallel hetero-tetramer (PDB accession 6EC0)^53^. Higher order assembly occurs through unit length filaments, into 10-nm fibres, which are directly comparable to SYCE2-TEX12 10-nm fibres, and are shown here for vimentin (middle). Scale bars, 100 nm. Reproduced from Helfand, et al. ^54^. The 10-nm IF fibres can further assemble into thick bundles, which are directly comparable with SYCE2-TEX12 40-nm fibres. Please see Kayser, et al. ^58^ for electron micrographs of keratin’s thick bundles.

The X-ray diffraction properties of SYCE2-TEX12 fibres are characteristic of the k-m-e-f family of fibrous α-proteins (keratin, myosin, epidermin and fibrinogen)^41,42^. These consist of 5.1 Å meridional arcs from coiled-coil repeats oriented along the fibrous axis, and equatorial reflections from lateral inter-helical spacings (9.5-9.8 Å and 12 Å for dimeric and three-/four-helical coiled-coils, respectively)^41,42^. Intermediate filaments (IF; including vimentin, lamin and keratin) are fibrous α-proteins that assemble from dimeric coiled-coil building blocks into laterally-associated tetramers, which interact end-to-end and laterally into 10-nm fibres (Extended Data Fig. 9)^47–54^. These stepwise assembly stages appear analogous to SYCE2-TEX12’s 2:2 building blocks, 4:4 clamped intermediates and 10-nm fibres. Further, IF proteins assemble through α-helical end motifs55, their 10-nm fibres form meshwork and bundled networks^56–58^, and lamin forms 3.5-nm fibres that are reminiscent of SYCE2-TEX12’s 4-nm fibres^47,59^. Thus, hierarchical SYCE2-TEX12 assembly bears striking resemblance to IF assembly (Extended Data Fig. 9).

The varied cellular functions of IF proteins can be attributed to their high tensile strength coupled with torsional and flexional freedom, which allows them to define axes, connect distant structures, form interaction hubs, and establish architectural meshworks that define cellular milieu^47–57,59^. What does this tell us about SYCE2-TEX12 function within the SC? Firstly, linear SYCE2-TEX12 fibres may establish a single continuous SC backbone that structurally underpins SC structure over micrometre distances, preventing disjunct and overlapping synapses, and providing a structural means for rapid signal transduction along the SC. This explains the presence of only short discontinuous stretches of nascent synapsis upon disruption of SYCE2 or TEX12^27,28^. Secondly, SYCE2-TEX12 fibres likely possess significant longitudinal strength, which could constrain the length of compacted synapsed meiotic chromosomes, whilst permitting substantial bending and torsional freedom during chromosomal dynamics and DNA exchanges of meiotic prophase and recombination^1^. Such flexion and torsional motions could be enhanced by gliding interactions of 10-nm fibres within bundled 40-nm fibres, in which a single fixed-length axis could be highly adaptive and responsive to external forces directed away from the longitudinal axis. Finally, SYCE2-TEX12 fibres could act as interaction hubs for formation of a meshwork assembly that defines SC architecture.

How do SYCE2-TEX12 fibres assemble with other SC components? The most prominent and conserved surface-exposed amino acids of the SYCE2-TEX12 structure are SYCE2 H89 and Y115, which mediate 4:4 assembly, and aromatic residues at the end-to-end assembly interface (including SYCE2 58-LYF-60 and TEX12 FFV). Thus, predicted interacting sites correspond to regions of the structural core that perform critical roles in fibrous self-assembly. Could interactions with other SC components directly regulate SYCE2-TEX12 assembly? Such interactions could trigger SYCE2-TEX12 fibre formation, or impose a specific SYCE2-TEX12 structure (such as by dictating the formation of only 2-nm rather than 4-nm fibres by binding to the SYCE2 tetramer interface) at key axial locations, such as recombination sites. This could mediate signalling, by propagating certain SYCE2-TEX12 fibrous conformations along the SC axis that impose function by regulating the local access of recombination factors. Similarly, phosphorylation events that are implicated in SC disruption are located at the N-terminal end of TEX12’s α-helical core^60^, and thereby in close proximity of SYCE2-TEX12 end-to-end assembly interfaces. Could post-translational modifications precipitate SC disassembly by directly disrupting SYCE2-TEX12 fibres? Further, SYCE2 and TEX12 have long unstructured termini, which contribute to assembly and may also contain protein binding sites. In this case, they may emanate from the fibrous surface – in a ‘bottle-brush’ conformation – to mediate cooperative interactions of SYCE2-TEX12 fibres with SC transverse filament and central element components. It thus remains a critical challenge to uncover how the SC’s major self-assembling systems of SYCP1, SYCP3 (and SYCP2-SYCP3) and SYCE2-TEX12 are bound together to achieve the enigmatic structure and function of the fully assembled SC.

## Methods

### Recombinant protein expression and purification

Sequences corresponding to regions of human SYCE2 (full-length, 1-218; core, 57-165; ΔS2C, 57-154) and TEX12 (full-length, 1-123; ΔCtip, 1-113; core, 49-123; core ΔCtip, 49-113) were cloned into pRSF-Duet1 (Novagen®) expression vectors for expression as TEV-cleavable N-terminal MBP- and His6-fusion proteins, respectively. Constructs were co-expressed in BL21 (DE3) cells (Novagen®), in 2xYT media, induced with 0.5 mM IPTG for 16 hours at 25°C. Cells were lysed by sonication in 20 mM Tris pH 8.0, 500 mM KCl, and fusion proteins were purified from clarified lysate through consecutive Ni-NTA (Qiagen), amylose (NEB) and HiTrap Q HP (GE Healthcare) ion exchange chromatography. Affinity tags were removed by incubation with TEV protease and cleaved samples were purified by HiTrap Q HP ion exchange chromatography and size exclusion chromatography (HiLoad™ 16/600 Superdex 200, GE Healthcare) in 20 mM Tris pH 8.0, 150 mM KCl, 2 mM DTT. Protein samples were concentrated using Pall Microsep™ Advance centrifugal devices, and were stored at -80°C following flash-freezing in liquid nitrogen. Protein samples were analysed by SDS-PAGE with Coomassie staining, and concentrations were determined by UV spectroscopy using a Cary 60 UV spectrophotometer (Agilent) with extinction coefficients and molecular weights calculated by ProtParam (http://web.expasy.org/protparam/).

### Crystallisation and structure solution of the SYCE2-TEX12 core ΔCtip 2:2 complex

SYCE2-TEX12 core ΔCtip protein crystals were obtained through vapour diffusion in sitting drops, by mixing 100 nl of 33 mg/ml protein at with 100 nl of crystallisation solution (35 % MPD) and equilibrating at 20°C for 4 months. Crystals were cryo-cooled in liquid nitrogen. X-ray diffraction data were collected at 0.9786 Å, 100 K, as 2000 consecutive 0.10° frames of 0.010 s exposure on a Pilatus3 6M detector at beamline I24 of the Diamond Light Source synchrotron facility (Oxfordshire, UK). Data were indexed, integrated in XDS61, scaled in *XSCAL*E62, and merged with anisotropic correction and cut-off level at a local I/σ(I) of 1.2 using the *STARANISO* server63. Crystals belong to monoclinic spacegroup P2_1_ (cell dimensions a = 88.52 Å, b = 24.19 Å, c = 88.48 Å, α = 90°, β = 115.7°, γ = 90°), with a 2:2 SYCE2-TEX12 heterotetramer in the asymmetric unit. Structure solution was achieved through fragment-based molecular replacement using *ARCIMBOLDO_LITE* ^64^, in which ten helices of 18 amino acids were placed by *PHASER* ^65^ and extended by tracing in *SHELXE* utilising its coiled-coil mode40. A correct solution was identified by a *SHELXE* correlation coefficient of 47.8%. Model building was performed through iterative re-building by *PHENIX Autobuild* ^66^ building in *COOT* ^67^. The structure was refined using *PHENIX refine* ^66^, using isotropic atomic displacement parameters with one TLS group. The structure was refined against data to anisotropy-corrected data with resolution limits between 2.42 Å and 3.36 Å, to *R* and *Rfree* values of 0.2871 and 0.3296 respectively, with 100% of residues within the favoured regions of the Ramachandran plot (0 outliers), clashscore of 6.89 and overall *MolProbity* score of 1.3868. The R-factors are higher than is typical for isotropic diffraction at this resolution, but are consistent with the combined effects of anisotropic diffraction and data modulation owing to the orientation of coiled-coils in parallel densely-packed arrays in which repetitive structures give rise to systematically strong and weak reflections within regions of reciprocal space^69^.

### Crystallisation and structure solution of the SYCE2-TEX12 core ΔCtip 4:4 complex

SYCE2-TEX12 core ΔCtip protein crystals were obtained through vapour diffusion in sitting drops, by mixing 100 nl of protein at 33 mg/ml with 100 nl of crystallisation solution (0.1 M sodium acetate, pH 4.0, 65 % MPD) and equilibrating at 20°C for 4 months. Crystals were cryo-cooled in liquid nitrogen. X-ray diffraction data were collected at 0.9688 Å, 100 K, as 2000 consecutive 0.10° frames of 0.020 s exposure on a Pilatus3 6M detector at beamline I24 of the Diamond Light Source synchrotron facility (Oxfordshire, UK). Data were processed using *AutoPROC* ^70^, in which indexing, integration and scaling were performed by *XDS* ^61^ and *XSCALE* ^62^, and anisotropic correction with a local I/σ(I) cut-off of 1.2 was performed by *STARANISO*63. Crystals belong to orthorhombic spacegroup P2_1_2_1_2 (cell dimensions a = 42.67 Å, b = 59.68 Å, c = 156.49 Å, α = 90°, β = 90°, γ = 90°), with a 2:2 SYCE2-TEX12 heterotetramer in the asymmetric unit. Structure solution was achieved by molecular replacement using *PHASER* ^65^, in which two copies of one end of the higher resolution SYCE2-TEX12 2:2 structure (PDB accession 6R17) were placed in the asymmetric unit. The structure was completed through manual building in *COOT* ^67^, with iterative refinement using *PHENIX refine* ^66^. The structure was refined against data to anisotropy-corrected data with resolution limits between 3.33 Å and 6.00 Å, to *R* and *Rfree* values of 0.3266 and 0.3672 respectively, with 100% of residues within the favoured regions of the Ramachandran plot (0 outliers), clashscore of 3.48 and overall *MolProbity* score of 1.1468. Similar to the 2:2 structure, the R-factors are higher than is typical for isotropic diffraction at this resolution but are consistent with the combined effects of anisotropic diffraction and data modulation in a fibrous coiled-coil lattice^69^.

### Crystallisation and X-ray diffraction analysis of SYCE2-TEX12 core ΔCtip fibres

SYCE2-TEX12 core ΔCtip protein crystals were obtained through vapour diffusion in hanging drops, by mixing 1 μl of protein at 17 mg/ml in buffer containing 20% glycerol with 1 μl of crystallisation solution (0.05 M caesium chloride, 0.1 M MES pH 6.5, 30% v/v Jeffamine M-600) and equilibrating at 20°C. Crystals were cryo-cooled in liquid nitrogen in crystallisation solution supplemented with 25% glycerol. X-ray diffraction data were collected at 0.9763 Å, 100 K, as individual 0.50° frames of 0.500 s exposure on a Pilatus3 6M detector at beamline I03 of the Diamond Light Source synchrotron facility (Oxfordshire, UK). Diffraction images were analysed using *Adxv.*

### Size-exclusion chromatography multi-angle light scattering (SEC-MALS)

The absolute molecular masses of SYCE2-TEX12 complexes were determined by size-exclusion chromatography multi-angle light scattering (SEC-MALS). Protein samples at >1 mg/ml were loaded onto a Superdex™ 200 Increase 10/300 GL size exclusion chromatography column (GE Healthcare) in 20 mM Tris pH 8.0, 150 mM KCl, 2 mM DTT, at 0.5 ml/min using an ÄKTA™ Pure (GE Healthcare). The column outlet was fed into a DAWN® HELEOS™ II MALS detector (Wyatt Technology), followed by an Optilab® T-rEX™ differential refractometer (Wyatt Technology). Light scattering and differential refractive index data were collected and analysed using ASTRA® 6 software (Wyatt Technology). Molecular weights and estimated errors were calculated across eluted peaks by extrapolation from Zimm plots using a dn/dc value of 0.1850 ml/g. SEC-MALS data are presented as differential refractive index (dRI) profiles, with fitted molecular weights (M_w_s) plotted across elution peaks.

### Circular dichroism (CD) spectroscopy

Far UV circular dichroism (CD) spectroscopy data were collected on a Jasco J-810 spectropolarimeter (Institute for Cell and Molecular Biosciences, Newcastle University). CD spectra were recorded in 10mM Na_2_HPO_4_/NaH_2_PO_4_ pH 7.5, at protein concentrations between 0.1-0.5 mg/ml, using a 0.2 mm pathlength quartz cuvette (Hellma), at 0.2 nm intervals between 260 and 185 nm at 4°C. Spectra were averaged across nine accumulations, corrected for buffer signal, smoothed and converted to mean residue ellipticity ([θ]) (x1000 deg.cm^2^.dmol^−1^.residue^−1^). Deconvolution was performed using the CDSSTR algorithm of the Dichroweb server (http://dichroweb.cryst.bbk.ac.uk)^71,72^. CD thermal denaturation was performed in 20 mM Tris pH 8.0, 150 mM KCl, 2 mM DTT, at protein concentrations between 0.1-0.4 mg/ml, using a 1 mm pathlength quartz cuvette (Hellma). Data were recorded at 222 nm, between 5°C and 95°C, at 0.5°C intervals with ramping rate of 2°C per minute, and were converted to mean residue ellipticity ([Θ_222_]) and plotted as % unfolded ([θ]_222,x_-[θ]_222,5_)/([θ]_222,95_-[θ]_222,5_). Melting temperatures (Tm) were estimated as the points at which samples are 50% unfolded.

### Electron Microscopy

Electron microscopy (EM) was performed using FEI Philips CM100 and 120 kV Hitachi HT7800 transmission electron microscopes at the Electron Microscopy Research Services, Newcastle University. Protein samples at 0.005-3 mg/ml were applied to carbon-coated grids and negative staining was performed using 2% (weight/volume) uranyl acetate. Images were analysed in the Fiji distribution of *ImageJ* ^73^, by applying background correction and an FFT Bandpass filter, and measuring the number, mean width and length of fibres using the Ridge detection algorithm. Data processing and statistical analysis were performed using *Prism* (GraphPad).

### Size-exclusion chromatography small-angle X-ray scattering (SEC-SAXS)

SEC-SAXS experiments were performed at beamline B21 of the Diamond Light Source synchrotron facility (Oxfordshire, UK). Protein samples at concentrations >5 mg/ml were loaded onto a Superdex™ 200 Increase 10/300 GL size exclusion chromatography column (GE Healthcare) in 20 mM Tris pH 8.0, 150 mM KCl at 0.5 ml/min using an Agilent 1200 HPLC system. The column outlet was fed into the experimental cell, and SAXS data were recorded at 12.4 keV, detector distance 4.014 m, in 3.0 s frames. Data were subtracted and averaged, and analysed for Guinier region *Rg* and cross-sectional *Rg* (*Rc*) using ScÅtter 3.0 (http://www.bioisis.net), and *P(r)* distributions were fitted using *PRIMUS* ^74^. *Ab initio* modelling was performed using *DAMMIF* ^75^, in which 30 independent runs were performed in P1 or P22 symmetry and averaged. Multi-phase SAXS *ab initio* modelling was performed using *MONSA* ^76^. Crystal structures and models were docked into *DAMFILT* and *MONSA* molecular envelopes using *SUPCOMB* ^77^, and were fitted to experimental data using *CRYSOL* ^78^ and *FoXS* ^79^, and flexible termini were modelled and fitted to experimental data using *CORAL* ^80^.

### Structural modelling

A heterodimeric coiled-coil of the SYCE2 and TEX12 C-termini, including their S2C and Ctip sequences (amino-acids 155-165 and 114-123, respectively) was modelled by *CCbuilder* 2.081 and was docked onto the 2:2 and 4:4 crystal structures using *PyMOL* Molecular Graphics System, Version 2.3.2 Schrödinger, LLC. The 4:4 ‘closed’ assembly was modelled by manual editing of C-terminal helical positioning in *COOT* ^67^, followed by iterations of energy minimisation using Rosetta Relax^82^ interspersed with idealisation by PHENIX geometry minimisation^66^. The 4:4 ‘open’ fibre assembly was modelled by imposing the crystallographic translation symmetry of 156 Å along the long axis onto 4:4 complexes with modelled C-terminal coiled-coils, followed by manual editing of C-terminal helical positioning in *COOT* ^67^, with iterations of energy minimisation using *Rosetta Relax* ^82^ interspersed with idealisation by *PHENIX* geometry minimisation^66^.

### Protein sequence and structure analysis

Multiple sequence alignments were generated using *Jalview* ^83^, and molecular structure images were generated using the *PyMOL* Molecular Graphics System, Version 2.0.4 Schrödinger, LLC.

## Extended Data

## Supplementary Material

Supplementary Table 1

## Figures and Tables

**Table 1:** Data collection, phasing and refinement statistics

	SYCE2-TEX12 ΔCtip 2:2 complex	SYCE2-TEX12 ΔCtip 4:4 assembly
**PDB accession**	6R17	6YQF

**Data collection**		
Space group	P2_1_	P2_1_2_1_2
Cell dimensions		
*a, b, c* (Å)	88.52, 24.19, 88.48	42.67, 59.68, 156.49
α, β γ (°)	90, 115.737, 90	90, 90, 90
Resolution (Å)	79.74 – 2.42 (2.74 – 2.42)*	47.45 – 3.33 (3.59 – 3.33)
Ellipsoidal resolution (Å)	2.416 (0.780 a* - 0.625 c*)	4.203 (a*)
(direction)	2.795 (b*)	3.710 (b*)
	2.926 (0.491 a* + 0.871 c*)	2.891 (c*)
*R*_meas_	0.074 (1.293)	0.246 (2.445)
*R*_pim_	0.049 (0.914)	0.098 (0.906)
*I* / σ(*I*)	10.2 (1.2)	4.7(1.0)
*CC_1/2_*	0.999 (0.564)	0.995 (0.516)
Completeness (spherical) (%)	63.2 (10.5)	64.8 (27.1)
Completeness (ellipsoidal) (%)	86.2 (51.3)	83.0 (82.7)
Redundancy	3.4 (3.0)	6.5 (7.2)

**Refinement**		
Resolution (Å)	79.74 – 2.42	47.45 – 3.33
No. reflections	8497	4053
*R*_work_ / *R*_free_	0.2871/0.3296	0.3266/0.3672
No. atoms	2536	2426
Protein	2526	2426
Ligand/ion	0	0
Water	10	0
*B*-factors	76.79	56.52
Protein	76.89	56.52
Ligand/ion	N/A	N/A
Water	53.44	N/A
R.m.s. deviations		
Bond lengths (Å)	0.005	0.001
Bond angles (°)	0.788	0.272

* Values in parentheses are for highest-resolution shell.

## Data Availability

Crystallographic structure factors and atomic co-ordinates have been deposited in the Protein Data Bank (PDB) under accession numbers 6R17 and 6YQF, and their corresponding raw diffraction images have been deposited at https://proteindiffraction.org/. The underlying data and uncropped gels corresponding to graphs and cropped gels in Figs. 1, 6 and 7, and Extended Data Fig. 3, are provided in Source Data files.

## References

[1] Zickler D, Kleckner N (2015). Recombination, Pairing, and Synapsis of Homologs during Meiosis. Cold Spring Harb Perspect Biol.

[2] Cahoon CK, Hawley RS (2016). Regulating the construction and demolition of the synaptonemal complex. Nat Struct Mol Biol.

[3] Kouznetsova A, Benavente R, Pastink A, Hoog C (2011). Meiosis in mice without a synaptonemal complex. PLoS One.

[4] Sanchez-Saez F (2020). Meiotic chromosome synapsis depends on multivalent SYCE1-SIX6OS1 interactions that are disrupted in cases of human infertility. Sci Adv.

[5] Geisinger A, Benavente R (2016). Mutations in Genes Coding for Synaptonemal Complex Proteins and Their Impact on Human Fertility. Cytogenet Genome Res.

[6] MacGregor IA, Adams IR, Gilbert N (2019). Large-scale chromatin organisation in interphase, mitosis and meiosis. Biochem J.

[7] Patel L (2019). Dynamic reorganization of the genome shapes the recombination landscape in meiotic prophase. Nat Struct Mol Biol.

[8] Martini E, Diaz RL, Hunter N, Keeney S (2006). Crossover homeostasis in yeast meiosis. Cell.

[9] Moses MJ (1956). Chromosomal structures in crayfish spermatocytes. J Biophys Biochem Cytol.

[10] Moses MJ (1968). Synaptinemal Complex. Annu Rev Genet.

[11] Westergaard M, von Wettstein D (1972). The synaptinemal complex. Annu Rev Genet.

[12] Solari AJ (1980). Synaptosomal complexes and associated structures in microspread human spermatocytes. Chromosoma.

[13] Spindler MC, Filbeck S, Stigloher C, Benavente R (2019). Quantitative basis of meiotic chromosome synapsis analyzed by electron tomography. Sci Rep.

[14] Solari AJ, Moses MJ (1973). The structure of the central region in the synaptonemal complexes of hamster and cricket spermatocytes. J Cell Biol.

[15] Schmekel K, Skoglund U, Daneholt B (1993). The three-dimensional structure of the central region in a synaptonemal complex: a comparison between rat and two insect species, Drosophila melanogaster and Blaps cribrosa. Chromosoma.

[16] Schucker K, Holm T, Franke C, Sauer M, Benavente R (2015). Elucidation of synaptonemal complex organization by super-resolution imaging with isotropic resolution. Proc Natl Acad Sci U S A.

[17] Dunce JM (2018). Structural basis of meiotic chromosome synapsis through SYCP1 self-assembly. Nat Struct Mol Biol.

[18] Yuan L (2002). Female germ cell aneuploidy and embryo death in mice lacking the meiosis-specific protein SCP3. Science.

[19] Yuan L (2000). The murine SCP3 gene is required for synaptonemal complex assembly, chromosome synapsis, and male fertility. Mol Cell.

[20] Yang F (2006). Mouse SYCP2 is required for synaptonemal complex assembly and chromosomal synapsis during male meiosis. J Cell Biol.

[21] Costa Y (2005). Two novel proteins recruited by synaptonemal complex protein 1 (SYCP1) are at the centre of meiosis. J Cell Sci.

[22] Schramm S (2011). A novel mouse synaptonemal complex protein is essential for loading of central element proteins, recombination, and fertility. PLoS Genet.

[23] Hamer G (2006). Characterization of a novel meiosis-specific protein within the central element of the synaptonemal complex. Journal of Cell Science.

[24] Gomez HL (2016). C14ORF39/SIX6OS1 is a constituent of the synaptonemal complex and is essential for mouse fertility. Nat Commun.

[25] de Vries FA (2005). Mouse Sycp1 functions in synaptonemal complex assembly, meiotic recombination, and XY body formation. Genes Dev.

[26] Bolcun-Filas E (2009). Mutation of the mouse Syce1 gene disrupts synapsis and suggests a link between synaptonemal complex structural components and DNA repair. PLoS Genet.

[27] Bolcun-Filas E (2007). SYCE2 is required for synaptonemal complex assembly, double strand break repair, and homologous recombination. Journal of Cell Biology.

[28] Hamer G (2008). Progression of meiotic recombination requires structural maturation of the central element of the synaptonemal complex. J Cell Sci.

[29] Fraune J, Schramm S, Alsheimer M, Benavente R (2012). The mammalian synaptonemal complex: Protein components, assembly and role in meiotic recombination. Exp Cell Res.

[30] Lu J (2014). Structural insight into the central element assembly of the synaptonemal complex. Sci Rep.

[31] Davies OR, Maman JD, Pellegrini L (2012). Structural analysis of the human SYCE2–TEX12 complex provides molecular insights into synaptonemal complex assembly. Open Biology.

[32] Syrjanen JL, Pellegrini L, Davies OR (2014). A molecular model for the role of SYCP3 in meiotic chromosome organisation. Elife.

[33] Syrjanen JL (2017). Single-molecule observation of DNA compaction by meiotic protein SYCP3. Elife.

[34] Dunne OM, Davies OR (2019). A molecular model for self-assembly of the synaptonemal complex protein SYCE3. J Biol Chem.

[35] Dunne OM, Davies OR (2019). Molecular structure of human synaptonemal complex protein SYCE1. Chromosoma.

[36] West AM (2019). A conserved filamentous assembly underlies the structure of the meiotic chromosome axis. Elife.

[37] Bollschweiler D (2019). Molecular architecture of the SYCP3 fibre and its interaction with DNA. Open Biol.

[38] Yuan L (1998). The synaptonemal complex protein SCP3 can form multistranded, cross-striated fibers in vivo. J Cell Biol.

[39] Ortiz R (2002). Cytochemical study of the distribution of RNA and DNA in the synaptonemal complex of guinea-pig and rat spermatocytes. Eur J Histochem.

[40] Caballero I (2018). ARCIMBOLDO on coiled coils. Acta Crystallogr D Struct Biol.

[41] Squire J (1981). The Structural Basis of Muscular Contraction.

[42] Er Rafik M, Doucet J, Briki F (2004). The intermediate filament architecture as determined by X-ray diffraction modeling of hard alpha-keratin. Biophys J.

[43] Bai Y, Luo Q, Liu J (2016). Protein self-assembly via supramolecular strategies. Chem Soc Rev.

[44] Garcia-Seisdedos H, Empereur-Mot C, Elad N, Levy ED (2017). Proteins evolve on the edge of supramolecular self-assembly. Nature.

[45] McManus JJ, Charbonneau P, Zaccarelli E, Asherie N (2016). The physics of protein self-assembly. Current Opinion in Colloid & Interface Science.

[46] Sandhu S (2019). A pseudo-meiotic centrosomal function of TEX12 in cancer. bioRxiv.

[47] Ahn J (2019). Structural basis for lamin assembly at the molecular level. Nat Commun.

[48] Aziz A (2012). The structure of vimentin linker 1 and rod 1B domains characterized by site-directed spin-labeling electron paramagnetic resonance (SDSL-EPR) and X-ray crystallography. J Biol Chem.

[49] Pang AH, Obiero JM, Kulczyk AW, Sviripa VM, Tsodikov OV (2018). A crystal structure of coil 1B of vimentin in the filamentous form provides a model of a high-order assembly of a vimentin filament. FEBS J.

[50] Chernyatina AA, Nicolet S, Aebi U, Herrmann H, Strelkov SV (2012). Atomic structure of the vimentin central alpha-helical domain and its implications for intermediate filament assembly. Proc Natl Acad Sci U S A.

[51] Lee CH, Kim MS, Chung BM, Leahy DJ, Coulombe PA (2012). Structural basis for heteromeric assembly and perinuclear organization of keratin filaments. Nat Struct Mol Biol.

[52] Bunick CG, Milstone LM (2017). The X-Ray Crystal Structure of the Keratin 1-Keratin 10 Helix 2B Heterodimer Reveals Molecular Surface Properties and Biochemical Insights into Human Skin Disease. J Invest Dermatol.

[53] Eldirany SA, Ho M, Hinbest AJ, Lomakin IB, Bunick CG (2019). Human keratin 1/10-1B tetramer structures reveal a knob-pocket mechanism in intermediate filament assembly. EMBO J.

[54] Helfand BT (2011). Vimentin organization modulates the formation of lamellipodia. Mol Biol Cell.

[55] Eldirany SA, Lomakin IB, Ho M, Bunick CG (2020). Recent insight into intermediate filament structure. Curr Opin Cell Biol.

[56] Koster S, Weitz DA, Goldman RD, Aebi U, Herrmann H (2015). Intermediate filament mechanics in vitro and in the cell: from coiled coils to filaments, fibers and networks. Curr Opin Cell Biol.

[57] Herrmann H, Aebi U (2016). Intermediate Filaments: Structure and Assembly. Cold Spring Harb Perspect Biol.

[58] Kayser J, Grabmayr H, Harasim M, Herrmann H, Bausch AR (2012). Assembly kinetics determine the structure of keratin networks. Soft Matter.

[59] Turgay Y (2017). The molecular architecture of lamins in somatic cells. Nature.

[60] Jordan PW, Karppinen J, Handel MA (2012). Polo-like kinase is required for synaptonemal complex disassembly and phosphorylation in mouse spermatocytes. J Cell Sci.

[61] Kabsch W (2010). Xds. Acta Crystallogr D Biol Crystallogr.

[62] Diederichs K, McSweeney S, Ravelli RB (2003). Zero-dose extrapolation as part of macromolecular synchrotron data reduction. Acta Crystallogr D Biol Crystallogr.

[63] Tickle IJ (2018). STARANISO.

[64] Rodriguez DD (2009). Crystallographic ab initio protein structure solution below atomic resolution. Nature Methods.

[65] McCoy AJ (2007). Phaser crystallographic software. Journal of Applied Crystallography.

[66] Adams PD (2010). PHENIX: a comprehensive Python-based system for macromolecular structure solution. Acta Crystallogr D Biol Crystallogr.

[67] Emsley P, Lohkamp B, Scott WG, Cowtan K (2010). Features and development of Coot. Acta Crystallogr D Biol Crystallogr.

[68] Chen VB (2010). MolProbity: all-atom structure validation for macromolecular crystallography. Acta Crystallographica Section D-Biological Crystallography.

[69] Thomas JMH, Keegan RM, Rigden DJ, Davies OR (2020). Extending the scope of coiled-coil crystal structure solution by AMPLE through improved ab initio modelling. Acta Crystallogr D Struct Biol.

[70] Vonrhein C (2011). Data processing and analysis with the autoPROC toolbox. Acta Crystallogr D Biol Crystallogr.

[71] Sreerama N, Woody RW (2000). Estimation of protein secondary structure from circular dichroism spectra: comparison of CONTIN, SELCON, and CDSSTR methods with an expanded reference set. Anal Biochem.

[72] Whitmore L, Wallace BA (2008). Protein secondary structure analyses from circular dichroism spectroscopy: methods and reference databases. Biopolymers.

[73] Schindelin J (2012). Fiji: an open-source platform for biological-image analysis. Nat Methods.

[74] Konarev PV, V VV, Sokolova AV, Koch MHJ, Svergun DI (2003). PRIMUS - a Windows-PC based system for small-angle scattering data analysis. J Appl Cryst.

[75] Franke D, Svergun DI (2009). DAMMIF, a program for rapid ab-initio shape determination in small-angle scattering. J Appl Crystallogr.

[76] Svergun DI (1999). Restoring low resolution structure of biological macromolecules from solution scattering using simulated annealing. Biophys J.

[77] Kozin MB, Svergun DI (2001). Automated matching of high- and low-resolution structural models. Journal of Applied Crystallography.

[78] Svergun DI, B C, K MHJ (1995). CRYSOL – a Program to Evaluate X-ray Solution Scattering of Biological Macromolecules from Atomic Coordinates. J Appl Cryst.

[79] Schneidman-Duhovny D, Hammel M, Tainer JA, Sali A (2016). FoXS, FoXSDock and MultiFoXS: Single-state and multi-state structural modeling of proteins and their complexes based on SAXS profiles. Nucleic Acids Res.

[80] Petoukhov MV (2012). New developments in the ATSAS program package for small-angle scattering data analysis. J Appl Crystallogr.

[81] Wood CW, Woolfson DN (2018). CCBuilder 2.0: Powerful and accessible coiled-coil modeling. Protein Sci.

[82] Nivon LG, Moretti R, Baker D (2013). A Pareto-optimal refinement method for protein design scaffolds. PLoS One.

[83] Waterhouse AM, Procter JB, Martin DM, Clamp M, Barton GJ (2009). Jalview Version 2--a multiple sequence alignment editor and analysis workbench. Bioinformatics.

