# Supplementary material for: Structural basis of meiotic chromosome synaptic elongation through hierarchical fibrous assembly of SYCE2-TEX12
Source: Nat Struct Mol Biol. Author manuscript; Available in PMC 2022 Feb 16. (PMC7612376; doi:10.1038/s41594-021-00636-z)
Supplement: Supplementary Table 1 [file EMS141971-supplement-Supplementary_Table_1.pdf]

Supplementary Table 1

Summary of SEC-SAXS data

**SYCE2-TEX12 core**	**WT 4:4**	**FFV 4:4**	**LFIL 2:2**	**ΔCtip 2:2**	**ΔS2C ΔCtip 2:2**	**WT 35 nm (l) fibre**	**WT 65 nm (l) fibre**
**Guinier analysis**							
I(0) (cm^{-1})	0.019 ±1.4x10^{-4}	0.120 ±2.0x10^{-4}	0.019 ±4.5x10^{-5}	0.044 ±1.3x10^{-4}	0.021 ±5.0x10^{-5}	0.031 ±7.4x10^{-4}	0.042 ±6.2x10^{-3}
Rg (Å)	49 ±0.33	49 ±1.16	47 ±0.59	49 ±0.31	41 ±1.69	86 ±1.30	167 ±25.3
Rc (Å)	19.4	19.4	12.4	12.2	11.7	16.6	15.3
q_{min} (Å^{-1})	0.056	0.0036	0.0070	0.0039	0.0043	0.0042	0.0035
***P(r)* analysis**							
I(0) (cm^{-1})	0.019 ±3.2x10^{-5}	0.122 ±2.0x10^{-4}	0.020 ±3.9x10^{-5}	0.044 ±1.1x10^{-4}	0.021 ±6.2x10^{-5}	0.031 ±6.3x10^{-4}	0.045 ±1.5x10^{-3}
Rg (Å)	53 ±0.13	52 ±0.12	51 ±0.13	50 ±0.19	45 ±0.20	93 ±2.05	183 ±3.9
D_{max} (Å)	190	188	180	185	154	350	650
Porod volume *(*Å^3)	138000	141000	86000	78400	65000	268000	264000
MW from Porod volume (kDa)	82	83	50	46	38	158	155
V_C *(*Å^2)	701	708	498	487	428	1030	1785
MW from V_C (kDa)	82	83	43	40	36	101	155
DAMMIF ab initio* modelling *(30 models)							
Symmetry	P22	P22	P1	P1	P1	N/A	N/A
NSD mean and s.d.	0.773 ±0.120	0.766 ±0.184	0.883 ±0.040	0.833 ±0.031	0.653 ±0.016	N/A	N/A
χ^2 (reference model)	1.33	1.18	1.15	1.31	0.99	N/A	N/A
MONSA* multi-phase *ab initio modelling							
Symmetry	P2	N/A	N/A	N/A	P2	N/A	N/A
χ^2	1.69	N/A	N/A	N/A	0.95-0.96	N/A	N/A
**Structural modelling**							
CRYSOL: Crystal structure (χ^2)	178	200	32.2	14.2	2.32	N/A	N/A
CRYSOL: + modelled C-terminal coiled-coil (χ^2)	13.5	20.2	8.84	3.40	1.23	N/A	N/A
CORAL: + modelled flexible N-termini (χ^2)	2.47	3.29	4.64	1.99	N/A	N/A	N/A
FoXS: fibril model (χ^2)	N/A	N/A	N/A	N/A	N/A	1.22	1.09
